# Context-dependent monoclonal antibodies against protein carbamidomethyl-cysteine

**DOI:** 10.1371/journal.pone.0242376

**Published:** 2020-11-24

**Authors:** Naw May Pearl Cartee, Soo Jung Lee, Simon G. Keep, Michael M. Wang

**Affiliations:** 1 Department of Neurology, University of Michigan, Ann Arbor, MI, United States of America; 2 Department of Veterans Affairs, Neurology Service, VA Ann Arbor Healthcare System, Ann Arbor, MI, United States of America; 3 Department of Molecular and Integrative Physiology, University of Michigan, Ann Arbor, MI, United States of America; Fondazione Pisana per la Scienza, ITALY

## Abstract

Protein sulfhydryl residues participate in key structural and biochemical functions. Alterations in sulfhydryl status, regulated by either reversible redox reactions or by permanent covalent capping, may be challenging to identify. To advance the detection of protein sulfhydryl groups, we describe the production of new Rabbit monoclonal antibodies that react with carbamidomethyl-cysteine (CAM-cys), a product of iodoacetamide (IAM) labeling of protein sulfhydryl residues. These antibodies bind to proteins labeled with IAM (but not N-ethylmaleimide (NEM) or acrylamide) and identify multiple protein bands when applied to Western blots of cell lysates treated with IAM. The monoclonal antibodies label a subset of CAM-cys modified peptide sequences and purified proteins (human von Willebrand Factor (gene:vWF), Jagged 1 (gene:JAG1), Laminin subunit alpha 2 (gene:LAMA2), Thrombospondin-2 (gene:TSP2), and Collagen IV (gene:COL4)) but do not recognize specific proteins such as Bovine serum albumin (gene:BSA) and human Thrombospondin-1 (gene:TSP1), Biglycan (gene:BGN) and Decorin (gene:DCN). Scanning mutants of the peptide sequence used to generate the CAM-cys antibodies elucidated residues required for context dependent reactivity. In addition to recognition of in vitro labeled proteins, the antibodies were used to identify selected sulfhydryl-containing proteins from living cells that were pulse labeled with IAM. Further development of novel CAM-cys monoclonal antibodies in conjunction with other biochemical tools may complement current methods for sulfhydryl detection within specific proteins. Moreover, CAM-cys reactive reagents may be useful when there is a need to label subpopulations of proteins.

## Introduction

Post-translation regulation of cysteine sulfhydryl groups impacts molecular function of a large fraction of proteins. In a majority of secreted proteins, cysteine thiols oxidize to form intramolecular disulfide bonds that play key roles in protein secondary structure and function. Disulfide bonds are reversible, and a series of redundant protein-dependent mechanisms exist in cells that enable isomerization of disulfides to promote proper protein folding [1]. Emerging evidence also supports regulated bidirectional conversion between disulfides and sulfhydryl residues that regulate protein function. Examples include hemostatic proteins such as tissue factor, Thrombospondin-1 (gene:TSP1), Von Willebrand factor (gene:vWF), Plasmin, T-cell surface glycoprotein CD4 (gene:CD4), and integrin subtypes, reviewed by [2–4], (Cytokine receptor common subunit gamma (gene:CD132) [5], and select intracellular proteins such as transcription factors of the Ets family [6]. Proteins are also subject to irreversible post translational modification at cysteine sulfhydryl residues, including nitrosylation that underlies important functional changes in a number of proteins across broad biological systems [7–9]. Unravelling the biological roles of cysteine thiols and disulfides in specific proteins has been made possible through chemical methods that differentiate cysteine states.

Multiple methods to detect sulfhydryl status have relied on covalent thiol-reactive reagents [10–14]. Frequently multiple thiol-reactive reagents are used in tandem to differentiate free thiols from those that participate in disulfide bonds. For example, free sulfhydryl residues can be labeled quantitatively with an initial reagent which is washed out and followed by reduction and reaction with a second reagent to target thiols occupied by reducible disulfide bonds. Using this approach, Metcalfe and colleagues performed mass spectrometry to identify labile sulfhydryl containing leukocyte proteins [15].

The most common thiol-trapping agents that react with cysteine sulfhydryl residues include N-ethylmaleimide (NEM) [16] and iodoacetamide (IAM) [17], which both form irreversible covalent products with proteins. Other reagents such as acrylamide have also been used to irreversibly label protein sulfhydryl residues. Recently, Holbrook et al [18] reported a monoclonal antibody, OX133, that recognizes protein covalently modified cysteine sulfhydryl residues after reaction with N-ethylmaleimide. OX133 was shown to be useful for detection of protein sulfhydryl residues by Western blotting, ELISA, and cell sorting. Expansion of immunological reagents that detect thiol modifications may offer opportunities to streamline thiol detection in complex systems that require tandem labeling of cysteines.

We now report the production of two novel antibodies which bind to cysteine sulfhydryl targets of IAM. We present experiments that characterize the target of these antibodies; the unique characteristics of the antibodies may complement current tools to track and quantify sulfhydryl residues in proteins.

## Methods

### Antibody generation preparation

All animal experiments were performed by Genscript. The immunization protocols were approved by the Genscript IACUC under animal protocol (ANT19-005; S1 Fig in S1 File). Methods of euthanasia were consistent with the recommendations of the Panel on Euthanasia of the American Veterinary Medical Association. A peptide antigen was synthesized corresponding to residues 253–278 (CFDPKYGEPKRPPNCARGSCPWDSQL) of human Myeloid associated differentiation marker like 2 (gene:MYADML2). The first amino acid, a reduced cysteine residue, was used as an anchor to conjugate the protein to adjuvant. CAM-cys residues were synthesized in place of the two remaining natural cysteine residues. Purity of the immunizing peptide synthesized at Genscript was verified by mass spectroscopy at Genscript. Rabbits were immunized followed by harvest of splenic cells that were fused to create hybridomas. ELISA on hybridoma supernatants was performed by coating plastic 96 well dishes with immunizing peptide at 1 ug/mL (0.1 mL per well) in PBS. Secondary antibodies against rabbit IgG Fc (HRP conjugate; GenScript A01856) was used in conjunction with chromogenic substrate read at 450nm. Positive clones that reacted by ELISA to the immunizing peptides at a titre of over 1:2000 (threshold >2-fold over negative media) were selected for further study. Antibodies used in the study were derived from hybridoma cell supernatants which were purified prior to use (stock concentrations were 134μg/mL [4E7] and 66μg/mL [52H11]).

### Chemicals, proteins, and molecular constructs

All chemicals were purchased from Sigma unless otherwise noted. Purified proteins were purchased from the following sources (all are human except where noted): Beta casein (bovine), Collagen I (gene:COL1), Collagen IV (Gene:COL4) (Sigma-Aldrich); Bovine serum albumin (gene:BSA) (bovine; New England BioLabs); Biglycan (gene:BGN), Decorin (gene:DCN), Jagged 1 (gene:JAG1), Interleukin 17 receptor C (gene:IL17RC), TSP1 and Thrombospondin-2 ((gene:TSP2) (R&D Systems)), Laminin subunit alpha 2 ((gene:LAMA2) (merosin; Millipore), vWF (Haematologic Technologies, Inc). Peptides were synthesized and verified by mass spectrometry by Thermo Fisher at >70% purity. Constructs used for mapping were generated based on the pEGFP-N1 (Clontech) backbone into which oligonucleotides encoding MYADML2 sequences were inserted using ligation into the multiple cloning site (Xho I and BamHI; The DNA sequences for the construct W, C1, C2, and Cb in Fig 7A are **CTCAGA**GCCACC***ATG***AAGCTTAAGTACGGTGAGCCCAAACGGCCCCCCAACTGTGCTCGGGGCAGCTGTCCCTGGGACAGCCAGCTGAG**GGATCC**, **CTCAGA**GCCACC***ATG***AAGCTTAAGTACGGTGAGCCCAAACGGCCCCCCAACTCCGCTCGGGGCAGCTGTCCCTGGGACAGCCAGCTGAG**GGATCC**, **CTCAGA**GCCACC***ATG***AAGCTTAAGTACGGTGAGCCCAAACGGCCCCCCAACTGTGCTCGGGGCAGCTCTCCCTGGGACAGCCAGCTGAG**GGATCC**, **CTCAGA**GCCACC***ATG***AAGCTTAAGTACGGTGAGCCCAAACGGCCCCCCAACTCCGCTCGGGGCAGCTCTCCCTGGGACAGCCAGCTGAG**GGATCC**, respectively. Both enzyme sites and the start codon are in bold and the mutation points were underscored.); the resulting amino acids added to GFP include residues contributed by the polylinker of the vector. Full coding sequences of the fusion proteins encoded by these constructs are listed in S2 Fig in S1 File.

### Cell culture and immunocytochemistry

HEK 293 cells were propagated on polystyrene dishes in DMEM supplemented with 10% fetal bovine serum, glutamine, and antibiotics (Invitrogen). Cells were grown in a 5% carbon dioxide environment at 37 ^o^C and routinely split using trypsin (Invitrogen). For protein analysis, 1x10^6^ cells were harvested in RIPA buffer (300μl) for total cell proteins and lysed by sonication (Branson Sonifier Cell Disruptor 200 output #4 pulsed mode, 50% duty cycle, 3–5 pulses, on ice) then centrifuged at 4°C for 20 minutes. 15μl of lysates were used for Western Blotting. For transfections, we mixed (1 μg) plasmid DNA with (3 μL) PolyJet (SignaGen, cat#SL100688) in 200 μL DMEM per manufacturer recommendations and added the mixture to cells in 6-well plates at 90% confluence. For protein analysis, we harvested cells for immunoblotting one day after transfection. Living cells were pulse labelled by adding IAM stock (100-fold higher than target concentration, in water) or an equivalent volume of water for two minutes. Media was removed after 2 minutes and replaced with fresh media without IAM. Cells were harvested for immunoblotting studies after 0, 8, 24, and 32 hours in media without IAM. For immunocytochemistry, we fixed cells with formalin for 30 minutes and washed with PBS three times. PBS with 2% BSA was used for blocking for 30 minutes and then antibodies at 1:50 in blocking buffer were applied for 2 hours. Secondary antibody (1:200 dilution in blocking buffer) was applied for 30 minutes followed by ABC staining (with Vector Elite ABC reagents) using diaminobenzidine (DAB) chromogenic detection. All washes were done three times with PBS.

### Protein alkylation

Peptide alkylation was performed in water at specified concentrations with stocks of IAM (100-fold higher relative to target concentration in water) for two hours at 37°C prior to application to membranes. When indicated, prior to alkylation, the purified protein was pre-reduced by 2.5 mM TCEP at 37°C for 30 minutes. Proteins were alkylated by mixing stocks of IAM, NEM, or acrylamide in water with total cell lysates or purified protein at 37°C (concentration and time specified for each experiment).

### Western and dot blotting

Proteins were separated on denaturing polyacrylamide gels (Invitrogen, 4–20% Tris Glycine) and transferred using an iBlot 2 system (Invitrogen, method P0 20V for 1 minute/23V for 4 minutes/25V for 2 minutes). Nitrocellulose immobilized proteins were blocked with 5% milk in TBST buffer and probed with antibodies in TBST buffer overnight at 4°C. Secondary antibody incubation was performed in TBST and room termperature for 30 minutes. All washes after primary and secondary antibody incubations were performed, for at least one minute, three times using TBST at room temperature. Antibody dilutions were 4E7 (1:1,000), 52H11 (1:1,000), OX133 (1:1,000; Absolute Antibody Ab00579-23.0 Rabbit IgG) and GFP (1:1,000; Santa Cruz Biotechnologies sc-9996). Secondary Ab info: Donkey anti-mouse IRDye 680RD (Li-Cor #926–68072, 1:10,000 dilution, AB_10953628) and Goat anti-rabbit IRDye 800CW (Li-Cor #926–32211, 1:10,000 dilution, AB_2651127). In some experiments, total protein was normalized by protein for tubulin with antibody E7 (1:666; DSHB). To quantify by Western blotting the percentage of labeled proteins during pulse chase labeling with alkylation agent, IAM, we calculated the total signal in each lane of the Western blots for CAM-cys, followed by correction of loading variation by probing for tubulin; cell lysate labeled with IAM at chase time 0 was set to 100%. Dot blots were performed by spotting peptides on nitrocellulose, followed by detection as before [19]. Briefly, peptides in 1 μL of water were spotted on membranes at concentrations listed and allowed to dry at room temperature. Membranes were moistened with TBST, and then probed without blocking with primary and secondary antibodies as described above for Western blots. Membranes were imaged using Li-Cor Odyssey Imager detection settings at 700 nm and 800 nm; Li-Core Image Studio software was used for data capture and quantification.

## Results

### mAbs that bind to iodoacetamide-modified proteins

Our original intent was to generate antibodies against MYADML2, a tetraspanin protein with multiple cysteine residues expressed in human brain [20]. Antigen analysis (https://www.genscript.com/peptide-antigen-database.html) indicated that an extracellular peptide containing three cysteines was a suitable immunogen. To enhance the potential for antibodies to bind to reduced protein on immunoblots, a peptide corresponding to this sequence (CFDPKYGEPKRPPNCARGSCPWDSQL; accession # NP_001138585.2) was generated with a reduced N-terminal anchoring cysteine and two CAM-cys residues in place of the remaining native cysteines. Rabbits were immunized against the synthetic peptide and hybridomas generated from cell fusion were screened for reactivity to the immunizing peptide.

**Fig 1:**
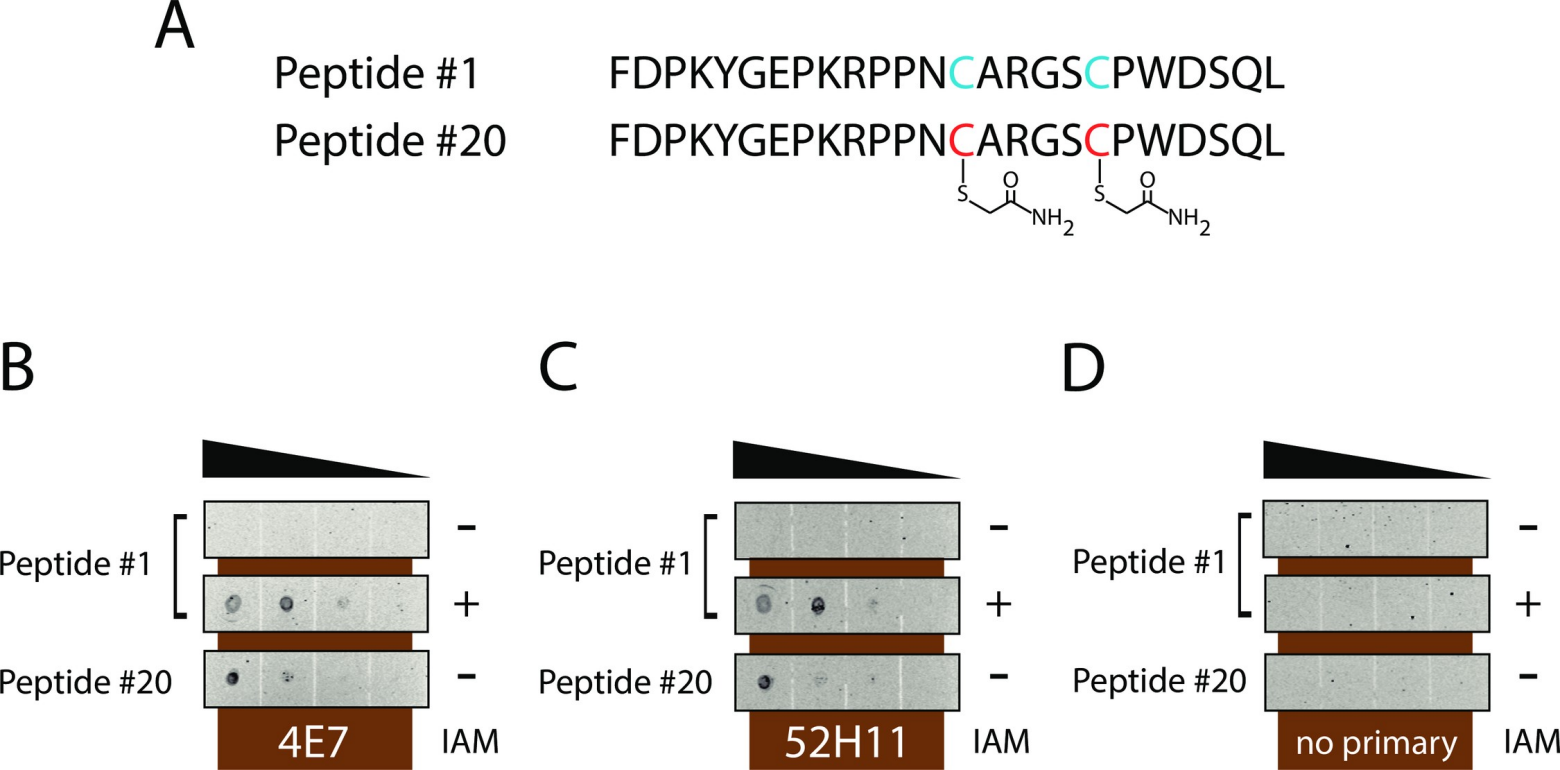
Two monoclonal antibodies recognize CAM-cys modified peptide sequences. (A) MYADML2 peptides used to screen for reactivity to modified cysteines lack the first anchoring cysteine present in the immunogen. Cysteine residues are highlighted in blue in peptide #1, which contains only natural amino acids. Peptide #20 was synthesized with two CAM-cys residues shown in red. An aliquot of peptide #1 was reacted with IAM (5 mM) for two hours at 37°C prior to application to membranes in the middle row of (B-D). One microliter dots of these peptides were applied to nitrocellulose at decreasing concentrations (1,000, 100, 10, and 1.0 μg/mL; denoted by triangles in B-D). Finally, membranes were probed with monoclonal antibodies 4E7 (B) and 52H11 (C) followed by secondary antibodies or with secondary antibodies alone (D) as a control.

Of the antibodies identified by ELISA, we found by dot blotting that two rabbit IgG monoclonals (4E7 and 52H11) bound to the CAM-cys modified MYADML2 peptides but were incapable of binding to peptides lacking CAM-cys modifications (peptide sequences shown in Fig 1A; blots shown in Fig 1B [probed with 4E7],1C [with 52H11], and 1D [with secondary antibody alone]). Furthermore, peptides synthesized with only natural residues that were subsequently reacted with IAM were recognized by the antibodies (compare first two rows in Fig 1B and 1C). Secondary antibodies did not bind to unmodified or IAM-modified peptides (Fig 1D).

**Fig 2:**
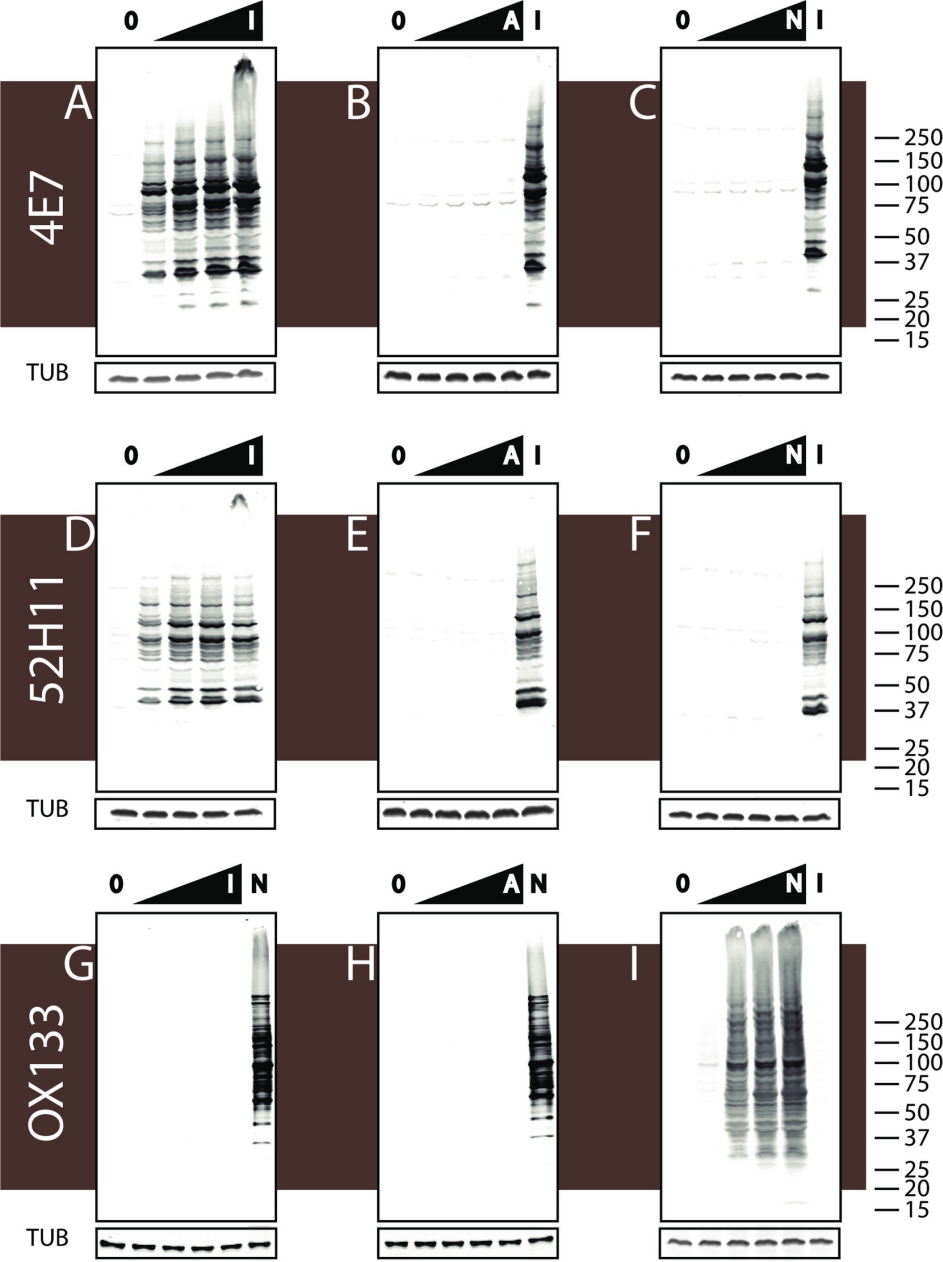
Two monoclonal antibodies recognize multiple proteins in cell lysates treated with IAM. Whole cell lysates from 293 cells were treated with increasing concentrations (0, 0.1, 0.5, 2, 5mM) of cysteine alkylating compounds IAM (A, D, G), acrylamide (B, E, H), and NEM (C, F, I). After Western blotting, panels of proteins were probed with 4E7 (A, B, C), 52H11 (D, E, F) or the NEM-cys targeted antibody OX133 (G, H, I). Lanes marked by 0 contain unmodified protein that serves as a baseline control. Labels in black triangles symbolize treatment dose dependent in IAM (I), acrylamide (A), and NEM (N). In B, C, E, F, and I, lane labeled I on the far right indicates lysates treated with IAM alone (2 mM). In G and H, the lane on the far right labeled with N contains lysates treated with NEM alone (2 mM). TUB = western blot for tubulin as a loading control.

We suspected that these MYADML2 non-reactive clones may, in fact produce, antibodies against CAM-cys that were incorporated in the immunizing peptide. To further test this, we prepared lysates from 293 cells and incubated them with a series of cysteine alkylating reagents at increasing concentrations (IAM [Fig 2A, 2D and 2G], acrylamide [Fig 2B, 2E and 2H], and NEM [Fig 2C, 2F and 2I]. The alkylated lysates were then analyzed by Western blotting with antibodies 4E7 and 52H11 which showed that antibody recognition of proteins increased in an IAM concentration dependent manner, whereas cysteine alkylation by NEM or acrylamide failed to induce antibody-reactive protein. Both antibodies consistently identified a series of bands of multiple molecular weights, suggesting that many IAM-labeled proteins from cell lysates could be recognized.

**Fig 3:**
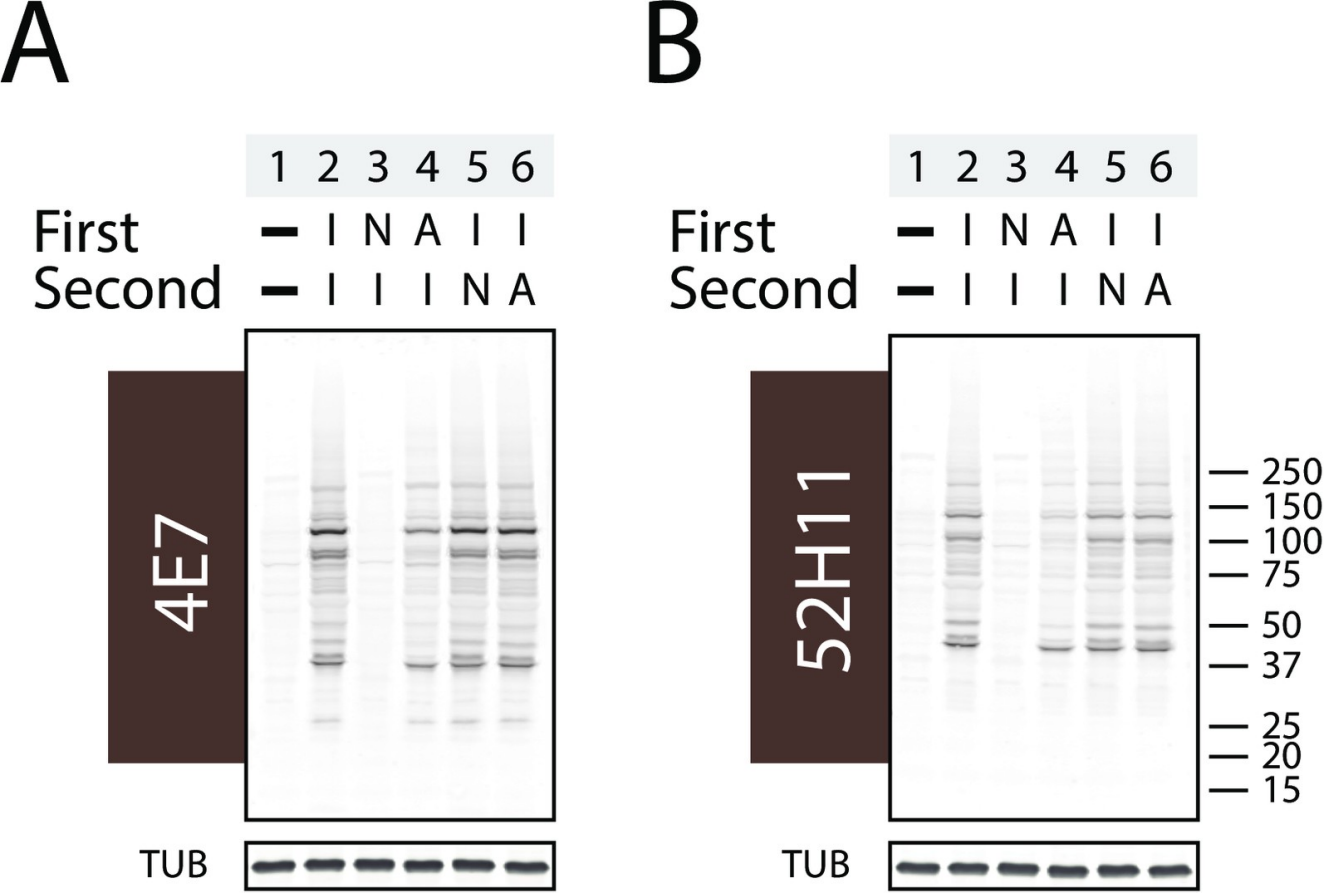
Effect of competitive blocking by cysteine alkylation on antibody binding to IAM-treated proteins. Potentially competitive cysteine alkylating agents were added either before or after IAM to determine whether cysteine modification could affect recognition of proteins incubated with IAM. Each sample of whole 293 cell lysate was sequentially exposed to different combinations of alkylators (labeled “First” then “Second”). The first lane of each panel was not treated with alkylators. The second lane was treated with IAM only. The third lane was treated first with NEM and then with IAM. The fourth lane was treated with acrylamide and then with IAM. The fifth lane was treated with IAM then NEM. And the sixth lane was treated with IAM followed by acrylamide. I = IAM (2mM); A = acrylamide (2mM); N = NEM (2mM). After Western blotting, membranes were probed with 4E7 (A) or 52H11 (B).

Competition experiments were performed to confirm the targets of IAM induced 4E7 (Fig 3A) and 52H11 reactivity (Fig 3B). As before, IAM incubation alone strongly increased antibody binding (compare lanes 1 and 2 of each blot). In contrast, incubation with NEM prior to IAM (third lane of each blot), reduced antibody-reactive protein on Western blots for both antibodies to baseline. Acrylamide incubation prior to IAM exposure (fourth lane of each blot) modestly reduced antibody-reactive protein on Western blots for both antibodies. Conversely, if NEM or acrylamide was incubated with lysates after IAM addition, there was no effect of protein reactivity (fifth and sixth lanes of each blot).

### Detection and tracking of sulfhydryl residues produced by living cells

**Fig 4:**
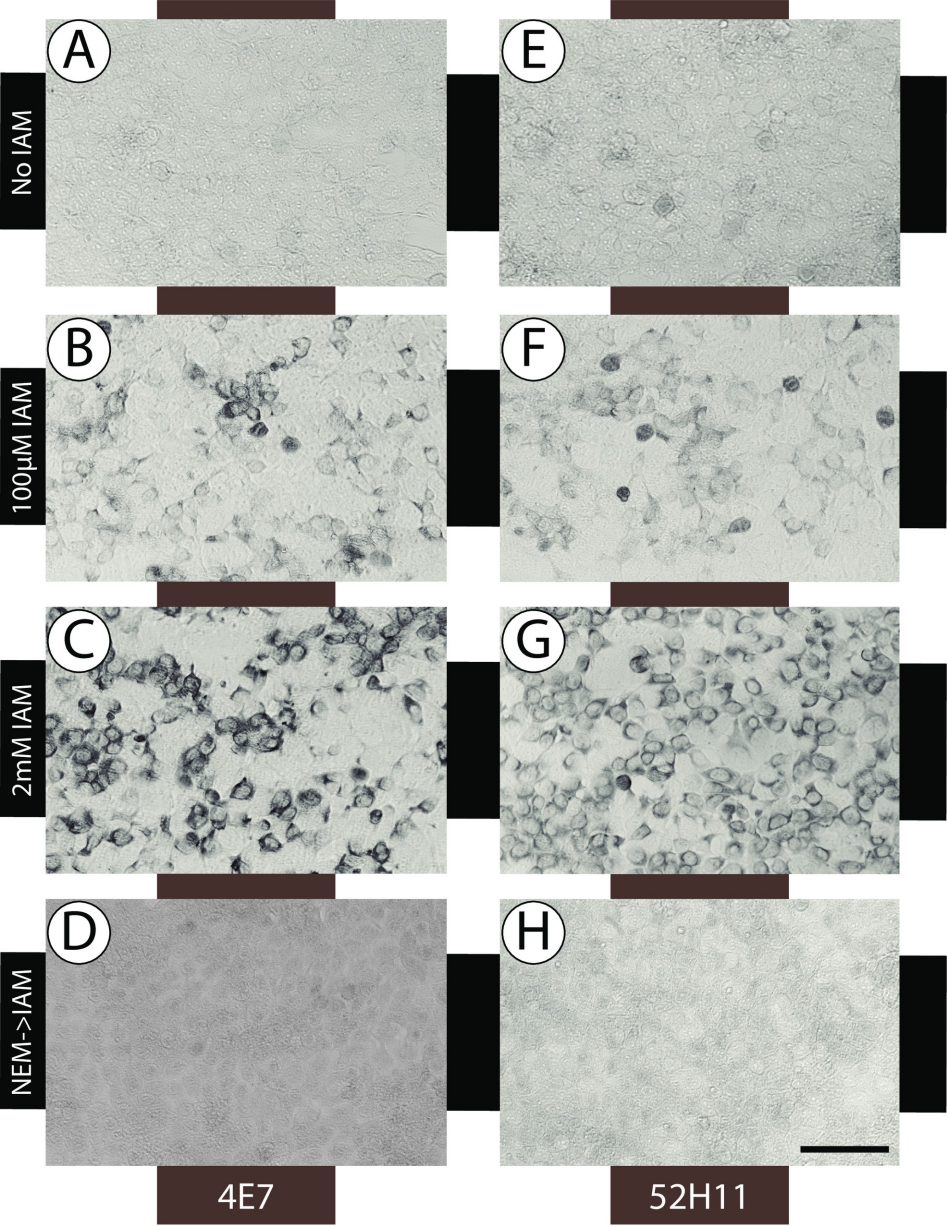
Detection of IAM labeled proteins from living cells. IAM was added to the growth media of 293 cells which were labeled at 37°C before fixation with formalin and staining with 4E7 (A-D) or 52H11 (E-H), all at 1:50 dilutions. The following IAM concentrations were applied for 10 minutes: None (A, E), 100μM (B, F), and 2mM (C, G). In some wells (D, H), NEM (100μM) was added to the media for 5 minutes before addition of IAM for 5 minutes.

We next investigated whether sulfhydryl residues in living cells could be detected using 4E7 and 52H11. Cells in culture were incubated with either IAM, or NEM followed by IAM, or an equivalent volume of water. After washing and fixation, cells were stained with 4E7 (Fig 4A–4D) and 52H11 (Fig 4E–4H). Unlabeled cells were not stained (Fig 4A and 4E). Both antibodies were capable of staining IAM-reacted cells proportional to the dose of IAM used. The signal was completely blocked by NEM pre-incubation (Fig 4D and 4H).

**Fig 5:**
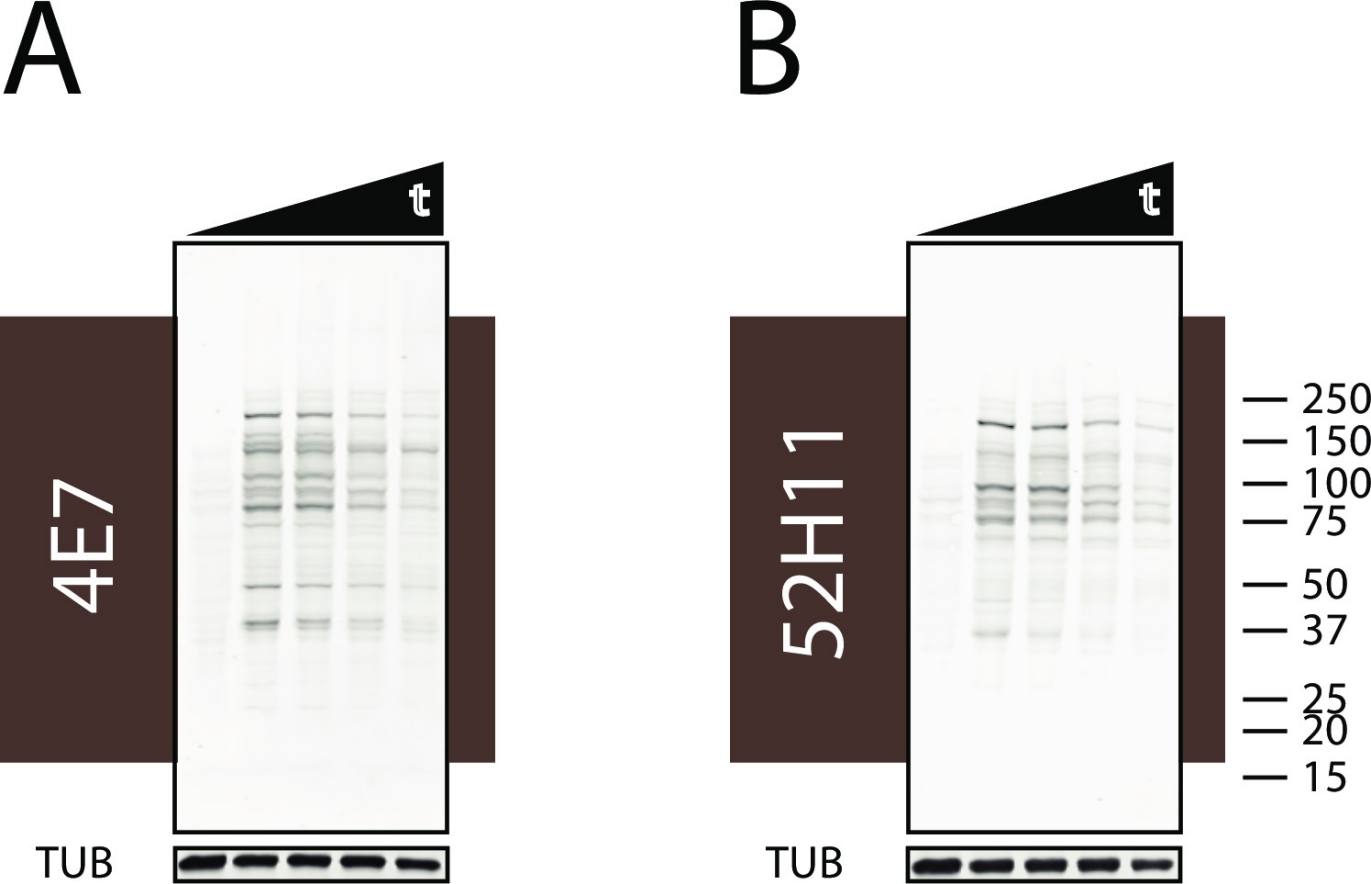
Detection of protein CAM-cys after pulse chase labeling of living cells with IAM. IAM (1mM) was added to growth media of 293 cells for 2 minutes followed by media without IAM for defined periods of time. Chase time intervals (lanes marked by triangle t) were 0, 8, 24, and 32 hours. Cells were collected for analysis of labeled protein content by Western blotting with 4E7 (A) and 52H11 (B). Western blotting for tubulin (TUB) served as a control for total protein loading.

IAM pulse labeling of living cells was performed to determine the lability of protein targets. After a short period of labeling, cells were incubated in media without IAM for defined chase periods. Cell lysates were then analyzed by Western blotting with 4E7 (Fig 5A) and 52H11 (Fig 5B). As before, a pulse of IAM in the culture media resulted in a broad spectrum of protein bands over a large array of molecular weights. After increasing chase periods, during which cultures were recovered in media without IAM, there was progressive attenuation of antibody reactivity with proteins. WB for tubulin showed that although similar amounts of protein were present during chase periods, the IAM labeled fraction was reduced over time. At 8, 24, and 32 hours, approximately 74, 41, and 34% of labeled protein remained for (A) and approximately 80, 45, and 37% of labeled protein remained for (B). These studies show the feasibility of using 4E7 and 52H11 to track the stability of sulfhydryl-labeled proteins in culture.

### Breadth of proteins detected by CAM-cys monoclonal antibodies

Both 4E7 and 52H11 detect ladders of protein on Western blots of populations of IAM treated cell lysates, suggesting that these affinity probes may recognize CAM-cys in multiple different proteins. To distinguish whether the antibodies recognize CAM-cys from all proteins versus selected proteins, we tested whether the antibodies were capable of binding to specific purified proteins after IAM-treatment.

**Fig 6:**
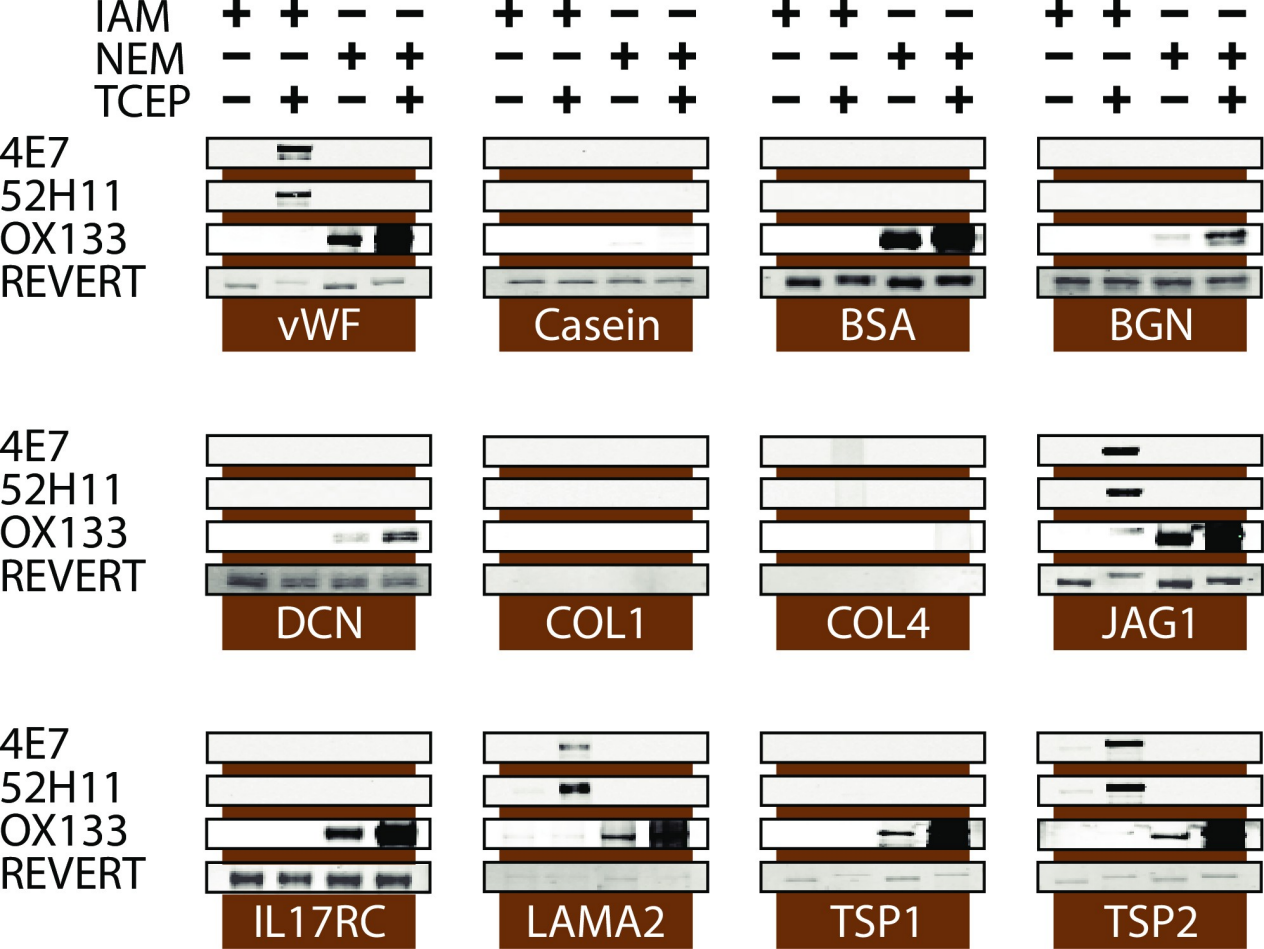
Antibodies to CAM-cys selectively target purified proteins. A series of purified proteins were treated in the presence or absence of the non-thiol reductant TCEP (2.5mM) with IAM or NEM (5mM). Proteins were then analyzed by Western blotting with 4E7, 52H11, or OX133. Selected CAM-cys labeled proteins reacted with 4E7 and 52H11. Equal loading of proteins was demonstrated by staining membranes with a non-selective protein stain (Revert, LI-COR). Proteins migrated at the expected molecular masses as follows (approximations): beta casein (27 kDa), BSA (65 kDa), BGN (40 kDa), DCN (38 kDa), COL1 (>250 kDa), COL4 (>250 kDa), JAG1 (170 kDa), IL17RC (75 kDa), LAMA2 (>180 kDa), vWF (260 kDa), TSP1 (150 kDa), TSP2 (155 kDa).

A series of purified, secreted proteins were treated with or without TCEP (Tris (2-carboxyethyl) phosphine) and then incubated with IAM or NEM to alkylate all available cysteines. Proteins were then analyzed by Western blotting with 4E7 and 52H11. Fig 6 shows that antibodies against CAM-cys specifically reacted with proteins treated with IAM only after treatment with TCEP (compare first and second lanes of each group). Furthermore, only a subset of the IAM-treated proteins was targeted by antibodies (vWF, JAG1, LAMA2, TSP2, and COL4); BSA, BGN, COL1, DCN, IL17RC, and TSP1 showed no detectable binding. In contrast, under the same reaction conditions, all cysteine-containing proteins except COL1 were recognized by OX133 after they were reduced and labeled with NEM, with an increased in reactivity after TCEP reduction (compare third and fourth lanes of each group). Casein, which does not contain cysteines, was not recognized by OX133 or CAM-cys antibodies under any conditions. These studies demonstrate the specificity and relative selectivity of CAM-cys antibodies for specific IAM-modified proteins.

### Sequence specificity of CAM-cys monoclonal antibodies

**Fig 7:**
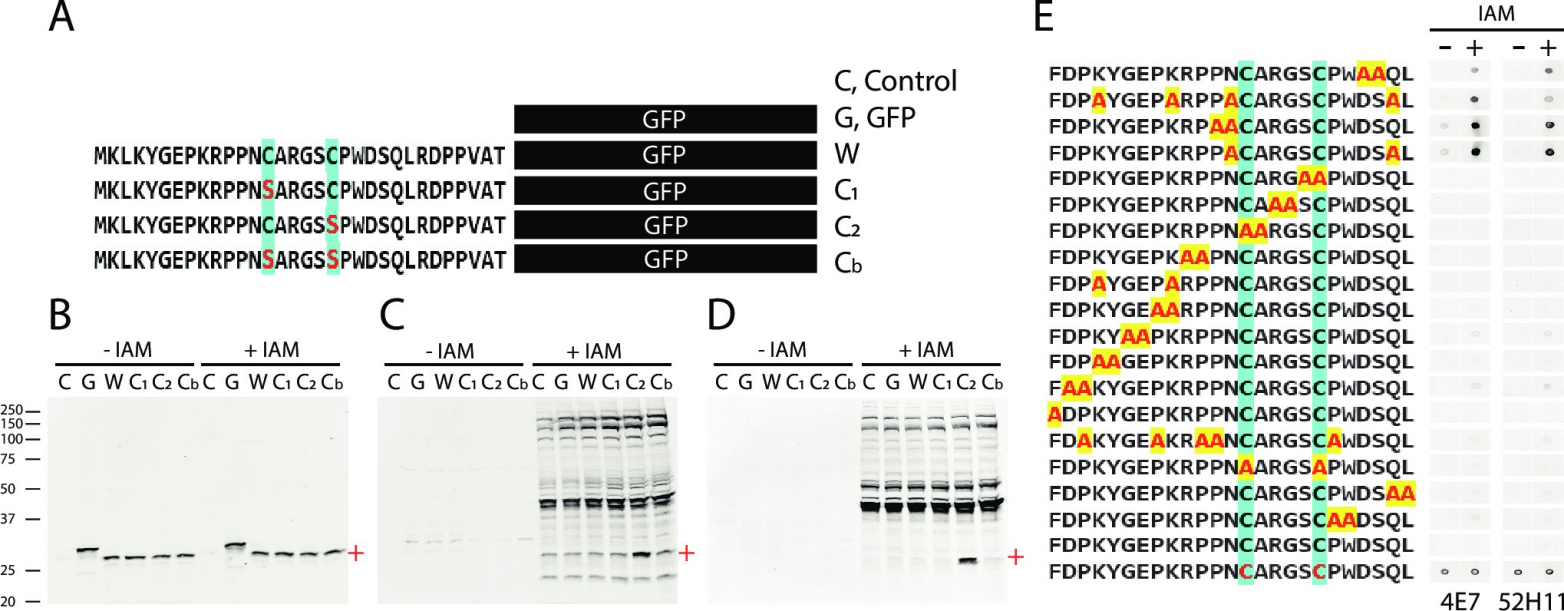
Peptide sequences targeted by 4E7 and 52H11. (A) Schematic of sequences fused to GFP in cDNA expression constructs. Wild type human MYADML2 fragment (W) and three variants with mutations in cysteines (to serine marked in red; C1, C2, and Cb) were transfected into cells. Lysates from transfectants were incubated at 37°C for 30 min with or without IAM (5 mM) were analyzed by GFP (B), 4E7 (C) and 52H11 (D) Western blotting. + indicates the molecular weight of peptide-fused GFP proteins. (E) a series of mutant peptides corresponding to the immunization epitope were analyzed by dot blotting. For IAM treated dots, peptides were incubated with IAM 5mM at 37°C for 30 minutes. Each dot contains 1 μg of each of the peptides shown, spotted on nitrocellulose.

To gain further insight into the sequence specificity of these antibodies, we generated recombinant clones that tagged the N-terminus of GFP with part of the sequence of the MYADML2 peptide used for immunization (Fig 7A). This peptide contains two cysteine residues. We also generated clones with site directed changes to each or both of these cysteines. We analyzed lysates from transfections of these constructs with and without reaction with iodoacetamide by immunoblotting. All lysates contain approximately the same amount of GFP (Fig 7B). Both 4E7 and 52H11 recognized proteins after IAM labeling (Fig 7C and 7D). The reagents were only able to recognize the GFP fusion in which the first cysteine of the MYADML2 peptide was intact and the second cysteine was mutated to serine. Antibodies did not react with GFP protein alone or with constructs where the first cysteine was mutated to serine. The construct with both cysteines intact did not react with antibodies. The antibodies to CAM-cys therefore appear to be very sensitive to single amino acid changes in the context of Western blotting.

A series of mutant peptides were also analyzed by dot blotting using 4E7 and 52H11 (Fig 7E). Peptides were spotted on filters without or with IAM reaction. IAM increased binding to most of the peptide sequences. Peptides that eliminated either cysteine of the target sequence were not recognized. Peptides that mutated the asparagine adjacent to the first cysteine increased binding, as did removal of acidic residues of the sequence. Several residues, adjacent to the first cysteine eliminated binding, irrespective of IAM incubation. This analysis reinforces that the monoclonal reagents recognize CAM-cys residues within specific contexts. It should be noted that a peptide with two synthetic CAM-cys residues was recognized by antibodies on dot blots, which was different from Western blot studies (lane W, with IAM, Fig 7C and 7D). In addition, mutation of either cysteine eliminated IAM-induced antibody binding on dot blots, though on Western blots, mutant C2 retained binding (lane C2, with IAM, Fig 7C and 7D). The differences are potentially due to the conformational constraints of expression with GFP compared to the unconstrained free peptide sequences.

## Discussion

We describe the production of two monoclonal antibodies, 4E7 and 52H11, that target protein CAM-cys residues, a product of thiol labeling with IAM. These antibodies detect IAM-modified proteins by Western blotting and immunocytochemistry in vitro and in cultured cells. Unlike other antibodies against modified cysteine residues, these antibodies recognize a subset of sulfhydryl-labeled proteins.

The antibodies described can be used in similar ways as OX133, an antibody developed against NEM modified protein sulfhydryl groups. The original description of OX133 suggests that it can be used to detect sulfhydryl residues in all proteins with cysteines (four of four tested) after labeling with NEM [18]. Our studies agree with the broad range of OX133 reactivity; all proteins containing cysteines that we tested exhibited OX133 reactivity after NEM incubation. However, the target range of 4E7 and 52H11 differ from OX133 in that the two CAM-cys reagents appear to react with only a subset of protein sulfhydryl residues. Competition experiments in which NEM or acrylamide, known cysteine reactive probes, were added prior to IAM, suggest that all residues targeted by IAM are also reactive with NEM. The subset of immunoreactive residues targeted by IAM were blocked with lower penetrance by acrylamide, potentially indicating that free thiols within cell lysates are incompletely targeted by acrylamide.

**Fig 8:**
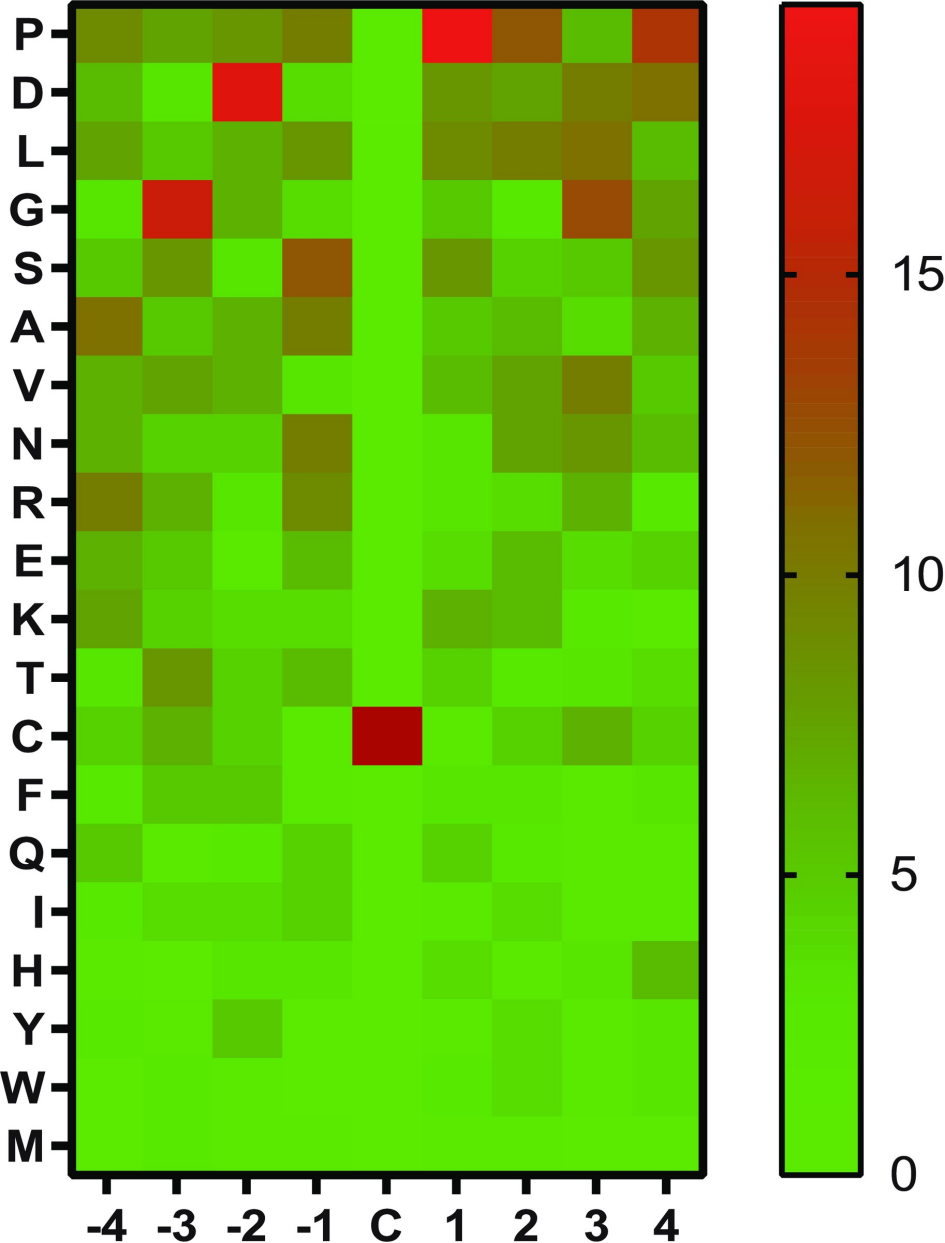
Sequences that do not react with CAM-cys antibodies. All cysteine residues from proteins that were found to be unreactive to both 4E7 and 52H11 after IAM treatment were analysed as follows: The four residues surrounding each side of the cysteines were arrayed for each of the eight positions relative to the cysteine. The relative frequency of each amino acid was then determined (percentage of all residues) and displayed by amino acid by position in a heat map. The most common amino acids at each position are listed at the top of the map.

Multiple lines of evidence suggest that 4E7 and 52H11 are selective for a subset of CAM-cys residues. Examination of 293 lysates shows that separable bands are identified by these antibodies, and only a fraction of purified proteins react with the antibodies. In more detailed studies, minor perturbations to the original MYADML2 target sequence fused to GFP affected antibody binding on Western blots. Only one of two potential CAM-cys residues was absolutely essential to binding; and, in fact, when both cysteine residues were converted to CAM-cys, there was elimination of binding on Western blots. Additional dot blot studies with synthetic peptides revealed the important residues adjacent to the first CAM-cys regulated binding, which further demonstrates the importance of sequence context. To provide initial insight into the sequence specificity of the new CAM-cys antibodies, we annotated the primary sequences surrounding all cysteines of proteins that did not contain any residues that reacted on Western blots (S3 Fig in S1 File). We included in this analysis of peptide sequences from Fig 7B that did not exhibit binding to either antibody and show the distribution of these sequences in Fig 8.

This analysis suggested that proline and aspartic acid residues close to cysteines were commonly found in non-binding sequences (top of Fig 8). Scanning peptide mutants (Fig 7E; first four sequences), demonstrated an increase in binding to CAM-cys peptides when an aspartic acid was changed to alanine and when an asparagine was mutated to alanine. In addition, aromatic ring structures were uncommon among sequences that did not bind antibodies after treatment with IAM (bottom of Fig 8). However, one must exercise caution in concluding that proline/aspartate decrease antibody affinity or that aromatic residues increase binding affinity, because other scanning mutants (Fig 7E) that affect these residues exert very little influence on binding levels. Thus, though the current study presents some information on the context that dictates binding preferences, definitive binding rules could not be determined, and binding to CAM-cys residues in other proteins is best determined experimentally.

We also show evidence that 4E7 and 52H11 antibodies are able to track the turnover of specific proteins in heterogeneous mixtures. In the future, Western blots of two-dimensional gel electrophoresis of cell extracts could be used to study protein dynamics of specific populations of sulfhydryl-containing proteins by quantitative analysis. To characterize sulfhydryl availability in specific proteins from complex protein mixes, changes in sulfhydryl states of proteins could be quantified by immunoprecipitation with target specific antibodies followed by IAM treatment and Western blotting with 4E7 and 52H11.

The reagents described here can also be used in combination with OX133 NEM antibodies to determine dynamic changes in sulfhydryl state using sequential labeling of cells. We show no NEM-induced cross reactivity of these antibodies, suggesting that the two sets of antibodies can be used to exclusively quantify NEM and IAM labeled residues.

Some potential limitations were discovered during the course of this study. First, in vivo labeling with IAM induces significant cell toxicity, at least in HEK293 cells. Thus, pulse chase studies can only be done in the short term with dense labeling, or only with low concentrations of IAM, which may limit labeling. Limited cell labeling is likely to preferentially highlight membrane proteins (see Fig 4), notwithstanding the purported cell permeability of IAM. It should be noted that both of the antibodies weakly recognize proteins that have not been treated with IAM on Western blotting, which mandates that use of the antibodies needs to employ a comparison of proteins with and without IAM. Finally, we attempted to use the monoclonal reagents to immunoprecipitate proteins, a necessary step in using the antibodies for mass spectroscopy-assisted proteomic analysis of proteins with reduced thiol groups. Though the antibodies were capable of enriching for CAM-cys containing proteins, we found an unacceptable amount of background adherence of CAM-cys proteins to beads which limited the utility of the reagents for this application under the conditions tested (S4 Fig in S1 File).

Despite these limits, our report provides the first proof of principle that 4E7 and 52H11 can be used to identify IAM-reacted sulfhydryl groups in subpopulations of proteins. In the future, new antibodies developed against distinct CAM-cys antigens or independent sulfhydryl-reactive probes [21–23] could be used for discrimination of sulfhydryl status of specific sequences.

## Supporting information

S1 File (PDF)

S2 File (PDF)
