# Supplementary material for: Context-dependent monoclonal antibodies against protein carbamidomethyl-cysteine
Source: PLoS One. 2020 Nov 24;15(11):e0242376. doi: 10.1371/journal.pone.0242376 (PMC7685443; doi:10.1371/journal.pone.0242376)

Supplemental Figure 1. Immunizations, cell fusion, and hybridoma growth parameters. All immunizations and hybridoma generation were performed by Genscript. Details regarding monoclonal antibody production provided by Genscript are summarized, including the immunization protocol (top) and cell manipulation and culture parameters (below).

Procedure	Schedule	Immune Dosage	Adjuvant	Site
Pre-immune bleed	T= -4 days			
Primary immunization	T= 0 day	200 µg /animal	Freund's Adjuvant, Complete	s.c. 4 points
Boost 1	T= 14 days	200 µg /animal	Freund's Adjuvant, Incomplete	s.c. 4 points
Test bleed 1	T= 21 days			
Boost 2	T= 35 days	200 µg /animal	Freund's Adjuvant, Incomplete	s.c. 4 points
Test bleed 2	T= 42 days			
Small scale purification after 3rd immunization	T= 42 days			
Joint evaluation	The antiserum and purified antibody were sent to client for tests to select the best animal for fusion			
Final boost	T= 95 days	400 µg /animal	No Adjuvant	i.p. and i.v.
Cell fusion (spleen isolated)	4 days after final boost			

Cell fusion and hybridoma culture

- Cell fusion partner was a murine line maintained at Genscript.
- Splenic cells and fusion partners were fused by Electro-fusion using protocols developed by Genscript.
- Hybridoma cell culture medium is DMEM with 10% FBS, 1X HT. Cells were cultured in 96-well plates.

Supplemental Figure 2. EGFP constructs used to map CAM-cys reactive sites in the MYADML2 sequence. For each construct used in Figure 7, we show two sequences 1) the open reading frames cloned into the EGFP expression vector specified in methods and 2) the predicted amino acid sequences of gene products expressed after transfection. EGFP sequences are in normal red. The start codon (**ATG**) is in bold red. Cysteine residues targeted by antibodies or their mutations to serine are highlighted in yellow or yellow on red, respectively. XbaI and BamHI cloning sites are shown in bold black (**CTCAGA** and **GGATCC**).

Construct W (wildtype MYADML2 peptide)

GCTAGCGCTACCGGA**CTCAGA**GCCACC**ATG**AAGCTTAAGTACGGTGAGCCCAAACGGCCCCCCAACTGTG
CTCGGGGCAGCTGTCCCTGGGACAGCCAGCTGAG**GGATCC**ACCGGTCGCCACCATGGTGAGCAAGGGCGA
GGAGCTGTTCACCGGGGTGGTGCCCATCCTGGTCGAGCTGGACGGCGACGTAAACGGCCACAAGTTCAGC
GTGTCCGGCGAGGGCGAGGGCGATGCCACCTACGGCAAGCTGACCCTGAAGTTCATCTGCACCACCGGCA
AGCTGCCCGTGCCCTGGCCCACCCTCGTGACCACCCTGACCTACGGCGTGCAGTGCTTCAGCCGCTACCC
CGACCACATGAAGCAGCACGACTTCTTCAAGTCCGCCATGCCCGAAGGCTACGTCCAGGAGCGCACCATC
TTCTTCAAGGACGACGGCAACTACAAGACCCGCGCCGAGGTGAAGTTCGAGGGCGACACCCTGGTGAACC
GCATCGAGCTGAAGGGCATCGACTTCAAGGAGGACGGCAACATCCTGGGGCACAAGCTGGAGTACAACTA
CAACAGCCACAACGTCTATATCATGGCCGACAAGCAGAAGAACGGCATCAAGGTGAACTTCAAGATCCGC
CACAACATCGAGGACGGCAGCGTGCAGCTCGCCGACCACTACCAGCAGAACACCCCCATCGGCGACGGCC
CCGTGCTGCTGCCCGACAACCACTACCTGAGCACCCAGTCCGCCCTGAGCAAAGACCCCAACGAGAAGCG
CGATCACATGGTCCTGCTGGAGTTCGTGACCGCCGCCGGGATCACTCTCGGCATGGACGAGCTGTACAAG
TACTCAGATCTCGAGCTCAAGCTTCGAATTCTGCAGTCGACGGTACCGCGGGCCCGGGATCCACCGGATC
TAGATAACTGA

MKLKYGEPKRPPNCARGSCPWDSQLRDPPVATMVSKGEELFTGVVPILVELDGDVNGHKF
SVSGEGEGDATYGKLTLKFICTTGKLPVPWPTLVTTLTYGVQCFSRYPDHMKQHDFFKSA
MPEGYVQERTIFFKDDGNYKTRAEVKFEGDTLVNRIELKGIDFKEDGNILGHKLEYNYNS
HNVYIMADKQKNGIKVNFKIRHNIEDGSVQLADHYQQNTPIGDGPVLLPDNHYLSTQSAL
SKDPNEKRDHMVLLEFVTAAGITLGMDELYKYSDLELKLRILQSTVPRARDPPDLDN*

Construct C1 (mutation of first MYADML2 cysteine to serine)

GCTAGCGCTACCGGA**CTCAGA**GCCACC**ATG**AAGCTTAAGTACGGTGAGCCCAAACGGCCCCCCAACTCCG
CTCGGGGCAGCTGTCCCTGGGACAGCCAGCTGAG**GGATCC**ACCGGTCGCCACCATGGTGAGCAAGGGCGA
GGAGCTGTTCACCGGGGTGGTGCCCATCCTGGTCGAGCTGGACGGCGACGTAAACGGCCACAAGTTCAGC
GTGTCCGGCGAGGGCGAGGGCGATGCCACCTACGGCAAGCTGACCCTGAAGTTCATCTGCACCACCGGCA
AGCTGCCCGTGCCCTGGCCCACCCTCGTGACCACCCTGACCTACGGCGTGCAGTGCTTCAGCCGCTACCC
CGACCACATGAAGCAGCACGACTTCTTCAAGTCCGCCATGCCCGAAGGCTACGTCCAGGAGCGCACCATC
TTCTTCAAGGACGACGGCAACTACAAGACCCGCGCCGAGGTGAAGTTCGAGGGCGACACCCTGGTGAACC
GCATCGAGCTGAAGGGCATCGACTTCAAGGAGGACGGCAACATCCTGGGGCACAAGCTGGAGTACAACTA
CAACAGCCACAACGTCTATATCATGGCCGACAAGCAGAAGAACGGCATCAAGGTGAACTTCAAGATCCGC
CACAACATCGAGGACGGCAGCGTGCAGCTCGCCGACCACTACCAGCAGAACACCCCCATCGGCGACGGCC
CCGTGCTGCTGCCCGACAACCACTACCTGAGCACCCAGTCCGCCCTGAGCAAAGACCCCAACGAGAAGCG
CGATCACATGGTCCTGCTGGAGTTCGTGACCGCCGCCGGGATCACTCTCGGCATGGACGAGCTGTACAAG

TACTCAGATCTCGAGCTCAAGCTTCGAATTCTGCAGTCGACGGTACCGCGGGCCCGGGATCCACCGGATC
TAGATAACTGA

MKLKYGEPKRPPN**S**ARGSCPWDSQLRDPPVATMVSKGEELFTGVVPILVELDGDVNGHKF
SVSGEGEGDATYGKLTLKFICTTGKLPVPWPTLVTTLTYGVQCFSRYPDHMKQHDFFKSA
MPEGYVQERTIFFKDDGNYKTRAEVKFEGDTLVNRIELKGIDFKEDGNILGHKLEYNYNS
HNVYIMADKQKNGIKVNFKIRHNIEDGSVQLADHYQQNTPIGDGPVLLPDNHYLSTQSAL
SKDPNEKRDHMVLLEFVTAAGITLGMDELYKYSDLELKLRILQSTVPRARDPPDLDN*

Construct C2 (mutation of second MYADML2 cysteine to serine)

GCTAGCGCTACCGGA**CTCAGA**GCCACC**ATG**AAGCTTAAGTACGGTGAGCCCAAACGGCCCCCCAACTGTG
CTCGGGGCAGCT**C**TCCCTGGGACAGCCAGCTGAG**GGATCC**ACCGGTCGCCACCATGGTGAGCAAGGGCGA
GGAGCTGTTCACCGGGGTGGTGCCCATCCTGGTCGAGCTGGACGGCGACGTAAACGGCCACAAGTTCAGC
GTGTCCGGCGAGGGCGAGGGCGATGCCACCTACGGCAAGCTGACCCTGAAGTTCATCTGCACCACCGGCA
AGCTGCCCGTGCCCTGGCCCACCCTCGTGACCACCCTGACCTACGGCGTGCAGTGCTTCAGCCGCTACCC
CGACCACATGAAGCAGCACGACTTCTTCAAGTCCGCCATGCCCGAAGGCTACGTCCAGGAGCGCACCATC
TTCTTCAAGGACGACGGCAACTACAAGACCCGCGCCGAGGTGAAGTTCGAGGGCGACACCCTGGTGAACC
GCATCGAGCTGAAGGGCATCGACTTCAAGGAGGACGGCAACATCCTGGGGCACAAGCTGGAGTACAACTA
CAACAGCCACAACGTCTATATCATGGCCGACAAGCAGAAGAACGGCATCAAGGTGAACTTCAAGATCCGC
CACAACATCGAGGACGGCAGCGTGCAGCTCGCCGACCACTACCAGCAGAACACCCCCATCGGCGACGGCC
CCGTGCTGCTGCCCGACAACCACTACCTGAGCACCCAGTCCGCCCTGAGCAAAGACCCCAACGAGAAGCG
CGATCACATGGTCCTGCTGGAGTTCGTGACCGCCGCCGGGATCACTCTCGGCATGGACGAGCTGTACAAG
TACTCAGATCTCGAGCTCAAGCTTCGAATTCTGCAGTCGACGGTACCGCGGGCCCGGGATCCACCGGATC
TAGATAACTGA

MKLKYGEPKRPPNCARGS**S**PWDSQLRDPPVATMVSKGEELFTGVVPILVELDGDVNGHKF
SVSGEGEGDATYGKLTLKFICTTGKLPVPWPTLVTTLTYGVQCFSRYPDHMKQHDFFKSA
MPEGYVQERTIFFKDDGNYKTRAEVKFEGDTLVNRIELKGIDFKEDGNILGHKLEYNYNS
HNVYIMADKQKNGIKVNFKIRHNIEDGSVQLADHYQQNTPIGDGPVLLPDNHYLSTQSAL
SKDPNEKRDHMVLLEFVTAAGITLGMDELYKYSDLELKLRILQSTVPRARDPPDLDN*

Construct Cb (mutation of both MYADML2 cysteines to serines)

GCTAGCGCTACCGGA**CTCAGA**GCCACC**ATG**AAGCTTAAGTACGGTGAGCCCAAACGGCCCCCCAACT**CC**G
CTCGGGGCAGCT**C**TCCCTGGGACAGCCAGCTGAG**GGATCC**ACCGGTCGCCACCATGGTGAGCAAGGGCGA
GGAGCTGTTCACCGGGGTGGTGCCCATCCTGGTCGAGCTGGACGGCGACGTAAACGGCCACAAGTTCAGC
GTGTCCGGCGAGGGCGAGGGCGATGCCACCTACGGCAAGCTGACCCTGAAGTTCATCTGCACCACCGGCA
AGCTGCCCGTGCCCTGGCCCACCCTCGTGACCACCCTGACCTACGGCGTGCAGTGCTTCAGCCGCTACCC
CGACCACATGAAGCAGCACGACTTCTTCAAGTCCGCCATGCCCGAAGGCTACGTCCAGGAGCGCACCATC
TTCTTCAAGGACGACGGCAACTACAAGACCCGCGCCGAGGTGAAGTTCGAGGGCGACACCCTGGTGAACC
GCATCGAGCTGAAGGGCATCGACTTCAAGGAGGACGGCAACATCCTGGGGCACAAGCTGGAGTACAACTA
CAACAGCCACAACGTCTATATCATGGCCGACAAGCAGAAGAACGGCATCAAGGTGAACTTCAAGATCCGC
CACAACATCGAGGACGGCAGCGTGCAGCTCGCCGACCACTACCAGCAGAACACCCCCATCGGCGACGGCC
CCGTGCTGCTGCCCGACAACCACTACCTGAGCACCCAGTCCGCCCTGAGCAAAGACCCCAACGAGAAGCG
CGATCACATGGTCCTGCTGGAGTTCGTGACCGCCGCCGGGATCACTCTCGGCATGGACGAGCTGTACAAG
TACTCAGATCTCGAGCTCAAGCTTCGAATTCTGCAGTCGACGGTACCGCGGGCCCGGGATCCACCGGATC
TAGATAACTGA

MKLKYGEPKRPPN**S**ARGS**S**PWDSQLRDPPVATMVSKGEELFTGVVPILVELDGDVNGHKF
SVSGEGEGDATYGKLTLKFICTTGKLPVPWPTLVTTLTYGVQCFSRYPDHMKQHDFFKSA
MPEGYVQERTIFFKDDGNYKTRAEVKFEGDTLVNRIELKGIDFKEDGNILGHKLEYNYNS
HNVYIMADKQKNGIKVNFKIRHNIEDGSVQLADHYQQNTPIGDGPVLLPDNHYLSTQSAL
SKDPNEKRDHMVLLEFVTAAGITLGMDELYKYSDLELKLRILQSTVPRARDPPDLDN*

Supplemental Figure 3. Cysteine adjacent sequences of antibody non-reactive proteins. The cysteine adjacent amino acid sequences (-4 to +4 from each cysteine) of the proteins that were not identified by both CAM-cys antibodies (4E7and 52H11). Biglycan (gene: BGN ACC# P21810), Fc-NTF (Fc protein fused to N-terminal fragment of Notch 3 protein, 1st EGF repeat). Decorin (gene: DCN ACC# P07585). Thrombospondin 1 (gene: TSP1 ACC # P07996-1). Interleukin 17 receptor C (gene:IL17RC ACC# NP_703190.1). Human IgG1 Fc Protein (gene: IGG1 ACC #P01857). Additional sequences were tested from synthetic peptides treated with IAM; the complete sequences were: Peptide #6 (FDPKYGEPKRPPNCAAASCPWDSQL), Peptide #7 (FDPKYGEPKRPPNAARGSCPWDSQL), Peptide #9 (FDPKYGEPKAAPNCARGSCPWDSQL) Peptide #18 (FDPAYGEPARPPNCARGSCPWDSQL). The results of analysis are shown in Figure 8 in the form of a heatmap showing the relative abundance of each amino acid at each of the 8 positions relative to the cysteine. N/A: Not applicable (no +4 position in sequence).

Protein	Sequence Number	-4	-3	-2	-1	C	+1	+2	+3	+4	Full 9 AA sequence
BGN	1	Y	S	A	M	**C**	P	F	G	C	YSAMCPFGC
	2	C	P	F	G	**C**	H	C	H	L	CPFGCHCHL
	3	F	G	C	H	**C**	H	L	R	V	FGCHCHLRV
	4	R	V	V	Q	**C**	S	D	L	G	RVVQCSDLG
	5	R	N	M	N	**C**	I	E	M	G	RNMNCIEMG
	6	V	N	D	F	**C**	P	M	G	F	VNDFCPMGF
	7	A	T	F	R	**C**	V	T	D	R	ATFRCVTDR
Fc-NTF	1	T	Q	N	P	**C**	P	P	L	K	TQNPCPPLK
	2	P	L	K	E	**C**	P	P	C	A	PLKECPPCA
	3	E	C	P	P	**C**	A	A	P	D	ECPPCAAPD
	4	P	M	V	T	**C**	V	V	V	D	PMVTCVVVD
	5	K	E	F	K	**C**	K	V	N	N	KEFKCKVNN
	6	F	S	L	T	**C**	M	I	T	G	FSLTCMITG
	7	S	L	F	A	**C**	S	V	V	H	SLFACSVVH
	8	L	A	P	P	**C**	L	D	G	S	LAPPCLDGS
	9	D	G	S	P	**C**	A	N	G	G	DGSPCANGG
	10	N	G	G	R	**C**	T	Q	L	P	NGGRCTQLP
	11	R	E	A	A	**C**	L	C	P	P	REAACLCPP
	12	A	A	C	L	**C**	P	P	G	W	AACLCPPGW
	13	V	G	E	R	**C**	Q	L	E	D	VGERCQLED
DCN	1	L	G	P	V	**C**	P	F	R	C	LGPVCPFRC
	2	C	P	F	R	**C**	Q	C	H	L	CPFRCQCHL
	3	F	R	C	Q	**C**	H	L	R	V	FRCQCHLRV
	4	R	V	V	Q	**C**	S	D	L	G	RVVQCSDLG
	5	S	S	D	F	**C**	P	P	G	H	SSDFCPPGH
	6	S	T	F	R	**C**	V	Y	V	R	STFRCVYVR
TSP1	1	L	M	H	V	**C**	G	T	N	R	LMHVCGTNR
	2	L	Y	I	D	**C**	E	K	M	E	LYIDCEKME
	3	R	N	K	G	**C**	S	S	S	T	RNKGCSSST
	4	L	Q	A	I	**C**	G	I	S	C	LQAICGISC
	5	C	G	I	S	**C**	D	E	L	S	CGISCGISC
	6	R	P	P	L	**C**	Y	H	N	G	RPPLCYHNG

	7	T	V	D	S	**C**	T	E	C	H	TVDSCTECH
	8	S	C	T	E	**C**	H	C	Q	N	SCTECHCQN
	9	T	E	C	H	**C**	Q	N	S	V	TECHCQNSV
	10	S	V	T	I	**C**	K	K	V	S	SVTICKKVS
	11	K	K	V	S	**C**	P	I	M	P	KKVSCPIMP
	12	P	I	M	P	**C**	S	N	A	T	PIMPCSNAT
	13	P	D	G	E	**C**	C	P	R	C	PDGECCPRC
	14	D	G	E	C	**C**	P	R	C	W	DGECCPRCW
	15	C	C	P	R	**C**	W	P	S	D	CCPRCWPSD
	16	E	W	T	S	**C**	S	T	S	C	EWTSCSTSC
	17	C	S	T	S	**C**	G	N	G	I	CSTSCGNGI
	18	R	G	R	S	**C**	D	S	L	N	RGRSCDSLN
	19	L	N	N	R	**C**	E	G	S	S	LNNRCEGSS
	20	Q	T	R	T	**C**	H	I	Q	E	QTRTCHIQE
	21	H	I	Q	E	**C**	D	K	R	F	HIQECDKRF
	22	P	W	S	S	**C**	S	V	T	C	PWSSCSVTC
	23	C	S	V	T	**C**	G	D	G	V	CSVTCGDGV
	24	R	I	R	L	**C**	N	S	P	S	RIRLCNSPS
	25	N	G	K	P	**C**	E	G	E	A	NGKPCEGEA
	26	E	T	K	A	**C**	K	K	D	A	ETKACKKDA
	27	K	K	D	A	**C**	P	I	N	G	KKDACPING
	28	P	I	D	G	**C**	L	S	N	P	PIDGCLSNP
	29	L	S	N	P	**C**	F	A	G	V	LSPNCFAGV
	30	A	G	V	K	**C**	T	S	Y	P	AGVKCTSYP
	31	G	S	W	K	**C**	G	A	C	P	GSWKCGACP
	32	K	C	G	A	**C**	P	P	G	Y	KCGACPPGY
	33	N	G	I	Q	**C**	T	D	V	D	NGIQCTDVD
	34	D	V	D	E	**C**	K	E	V	P	DVDECKEVP
	35	V	P	D	A	**C**	F	N	H	N	VPDACFNHN
	36	G	E	H	R	**C**	E	N	T	D	GEHRCENTD
	37	P	G	Y	N	**C**	L	P	C	P	PGYNCLPCP
	38	N	C	L	P	**C**	P	P	R	F	NCLPCPPRF
	39	N	K	Q	V	**C**	K	P	R	N	NKQVCKPRN
	40	P	R	N	P	**C**	T	D	G	T	PRNPCTDGT
	41	G	T	H	D	**C**	N	K	N	A	GTHDCNKNA
	42	K	N	A	K	**C**	N	Y	L	G	KNAKCNYLG
	43	P	M	Y	R	**C**	E	C	K	P	PMYRCECKP
	44	Y	R	C	E	**C**	K	P	G	Y	YRCECKPGY
	45	N	G	I	I	**C**	G	E	D	T	NGIICGEDT
	46	E	N	L	V	**C**	V	A	N	A	ENLVCVANA
	47	A	T	Y	H	**C**	K	K	D	N	ATYHCKKDN
	48	K	K	D	N	**C**	P	N	L	P	KKDNCPNLP
	49	I	G	D	A	**C**	D	D	D	D	IGDACDDDD
	50	D	R	D	N	**C**	P	F	H	Y	DRDNCPFHY
	51	V	G	D	R	**C**	D	N	C	P	VGDRCDNCP
	52	R	C	D	N	**C**	P	Y	N	H	RCDNCPYNH

	53	E	G	D	A	**C**	A	A	D	I	EGDACAADI
	54	E	R	D	N	**C**	G	Y	V	Y	ERDNCGYVY
	55	V	G	D	Q	**C**	D	N	C	P	VGDQCDNCP
	56	Q	C	D	N	**C**	P	L	E	H	QCDNCPLEH
	57	I	G	D	T	**C**	D	N	N	Q	IGDTCDNNQ
	58	N	L	D	N	**C**	P	Y	V	P	NLDNCPYVP
	59	K	G	D	A	**C**	D	H	D	D	KGDACDHDD
	60	D	K	D	N	**C**	R	L	V	P	DKDNCRLVP
	61	R	G	D	A	**C**	K	D	D	F	RGDACKDDF
	62	I	D	D	I	**C**	P	E	N	V	IDDICPENV
	63	Q	T	V	N	**C**	D	P	G	L	QTVMCDPGL
	64	L	K	Y	E	**C**	R	D	P	N/A	LKYECRDP no +4
IL17RC	1	D	A	T	H	**C**	S	P	G	L	DATHCSPGL
	2	P	G	L	S	**C**	R	L	W	D	PGLSCRLWD
	3	S	D	I	L	**C**	L	P	G	D	SDILCLPGD
	4	L	V	L	R	**C**	Q	K	E	T	LVLRCQKET
	5	K	E	T	D	**C**	D	L	C	L	KETDCDLCL
	6	D	C	D	L	**C**	L	R	V	A	DCDLCLRVA
	7	P	T	A	R	**C**	V	L	L	E	PTARCVLLE
	8	V	V	Y	D	**C**	F	E	A	A	VVYDCFEAA
	9	Q	L	P	D	**C**	R	G	L	E	QLPDCRGLE
	10	S	I	P	S	**C**	W	A	L	P	SIPSCWALP
	11	D	L	V	P	**C**	L	C	I	Q	DLVPCLCIQ
	12	V	P	C	L	**C**	I	Q	V	W	VPCLCIQVW
	13	R	T	N	I	**C**	P	F	R	E	RTNICPFRE
	14	L	D	A	P	**C**	S	L	P	A	LDAPCSLPA
	15	E	A	A	L	**C**	W	R	A	P	EAALCWRAP
	16	G	G	D	P	**C**	Q	P	L	V	GGDPCQPLV
	17	H	P	N	L	**C**	V	Q	V	N	HPNLCVQVN
	18	Q	L	Q	E	**C**	L	W	A	D	QLQECLWAD
	19	N	R	S	L	**C**	A	L	E	P	NRSLCALEP
	20	E	P	S	G	**C**	T	S	L	P	EPSGCTSLP
	21	Q	S	G	Q	**C**	L	Q	L	W	QSGQCLQLW
	22	A	L	W	A	**C**	P	M	D	K	ALWACPMDK
	23	V	W	L	A	**C**	L	L	F	A	VWLACLLFA
	24	A	S	A	L	**C**	Q	L	P	L	ASALCQLPL
	25	A	V	A	L	**C**	S	E	W	L	AVALCSEWL
	26	A	S	L	S	**C**	V	L	P	D	ASLSCVLPD
	27	Y	V	G	A	**C**	F	D	R	L	YVGACFDRL
IgG1	1	A	A	L	G	**C**	L	V	K	D	AALGCLVKD
	2	Q	T	Y	I	**C**	N	V	N	H	QTYOCNVNH
	3	E	P	K	S	**C**	D	K	T	H	EPKSCDKTH
	4	K	T	H	T	**C**	P	P	C	P	KTHTCPPCP
	5	T	C	P	P	**C**	P	A	P	E	TCPPCPAPE
	6	P	E	V	T	**C**	V	V	V	D	PEVTCVVVD
	7	K	E	Y	K	**C**	K	V	S	N	KEYKCKVSN

	8	V	S	L	T	**C**	L	V	K	G	VSLTCLVKG
	9	N	V	F	S	**C**	S	V	M	H	NVFSCSVMH
Peptide #6	1	R	P	P	N	**C**	A	A	A	G	RPPNCAAAG
	2	A	A	G	S	**C**	P	W	D	S	AAGSCPWDS
Peptide #7	1	A	R	G	S	**C**	P	W	D	S	ARGSCPWDS
Peptide #9	1	A	A	P	N	**C**	A	R	G	S	AAPNCARGS
	2	A	R	G	S	**C**	P	W	D	S	ARGSCPWDS
Peptide #18	1	R	P	P	N	**C**	A	R	G	S	RPPNCARGS
	2	A	R	G	S	**C**	P	W	D	S	ARGSCPWDS

Supplemental Figure 4. Identifying protein-IAM conjugates by immunoprecipitation. HEK293 cells lysate (in RIPA buffer) or vWF protein (in PBS, pre-reduced in 2.5mM TCEP at 37°C for 30 minutes) were incubated with 5mM IAM (iodoacetamide) for 3hr at 37°C. To remove excess IAM, samples were then dialyzed in Slide-A-Lyzer Mini dialysis devices (10K MWCO, Thermo Scientific) for 20 to 24 hours at 4°C against PBS. Dialyzed samples were then incubated with either RIPA or 2 ug purified CAM-cys monoclonal antibodies (4E7 or 52H11) for 18 to 20 hours at 4°C. Protein A agarose beads (Millipore) which were pre-blocked with 1% BSA were used for immunoprecipitation of proteins. HEK293 cells lysates and vWF without conjugation with IAM were included as controls. (A) shows that the 4E7 (lane 4) and 52H11 (lane 6) were able to pull down HEK293 protein-IAM conjugates, but IAM-treated HEK293 lysates pulled down without antibody also captured many protein bands (lane 2). One unique protein band observed around 10kD was increased in immunoprecipitates prepared with 4E7 or 52H11 (see lanes 4 and 6, band marked with red dot) compared to samples pulled down without antibody (lane 2). A protein smear was also observed above 250kD in the IAM conjugated samples immunoprecipitated by 4E7 (lane 4) or 52H11 (lane 6), which are marked with the red vertical line. The antibodies mixed with lysates without IAM were applied to lane 3 (4E7) and lane 5 (52H11). Immunoblotting for tubulin as a negative control shows bands in all six lanes, but more tubulin was detected in IP from samples conjugated with IAM (compare lane 1 to lane 2). This indicated that tubulin association with beads is increased by IAM labeling. (B) Purified vWF was also used for immunoprecipitation by 4E7 and 52H11. This shows similar results to HEK293 lysates in that both 4E7 (lane 4) and 52H11 (lane 6) precipitates included vWF conjugated with IAM (see red dots). A lighter band was also observed in the IP sample containing vWF conjugated with IAM without antibodies (lane2), suggesting that a small amount of vWF treated with IAM binds directly to beads. The antibodies mixed with vWF without IAM were applied to lane 3 (4E7) and lane 5 (52H11). SDS-PAGE was carried out after boiling the samples for 3 minutes in the sample loading buffer containing reducing agent.

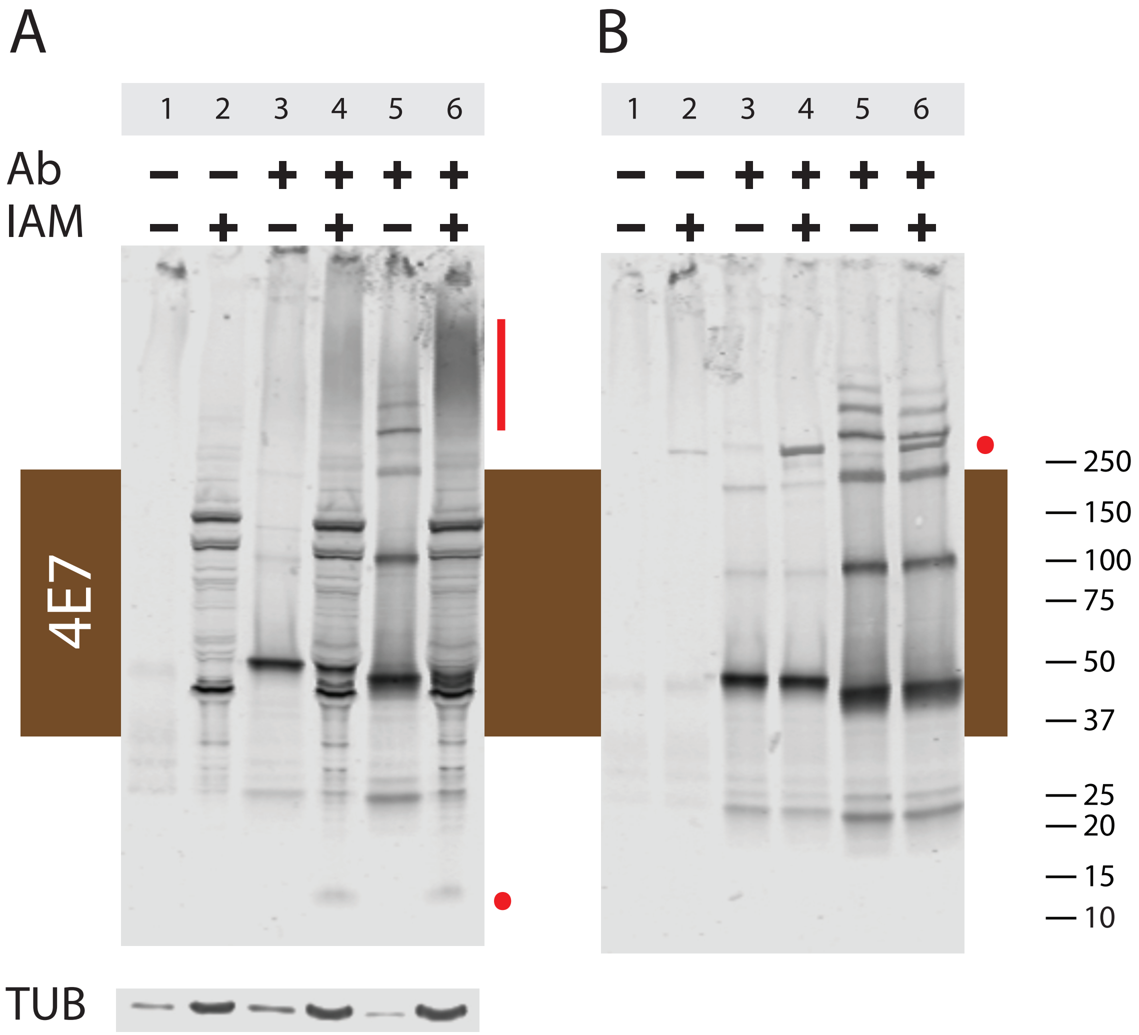

Supplement: S1 File — (PDF) [file pone.0242376.s001.pdf]
