# Supplementary material for: Context-dependent monoclonal antibodies against protein carbamidomethyl-cysteine
Source: PLoS One. 2020 Nov 24;15(11):e0242376. doi: 10.1371/journal.pone.0242376 (PMC7685443; doi:10.1371/journal.pone.0242376)
Supplement: S2 File — (PDF) [file pone.0242376.s002.pdf]

Figure 2

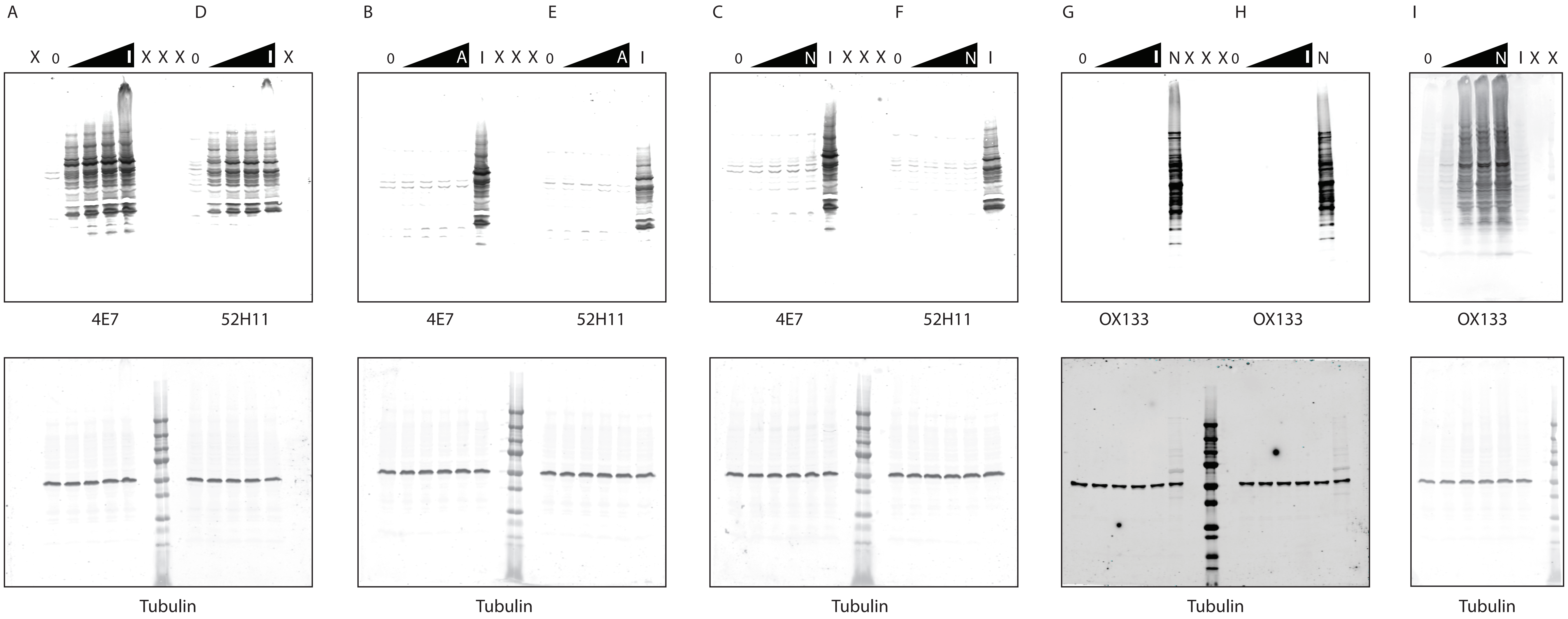

Samples were loaded as indicated in the figures above and Odyssey LICOR imager was used for image capture.
0 = no treatment I = Iodoacetamide A = Acrylamide N = N-Ethylmaleimide X = samples not included in the manuscript figures.

Figure 3

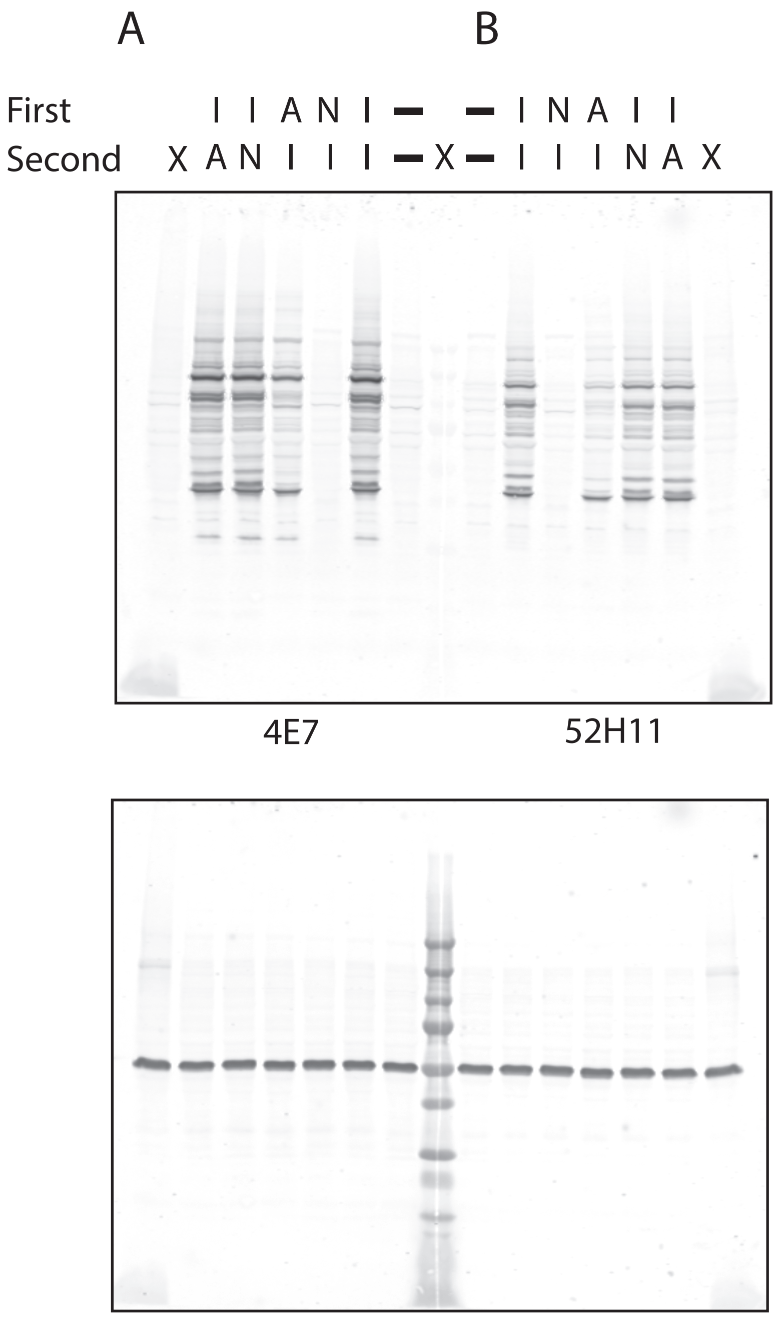

Samples were loaded as indicated in the figures and Odyssey LICOR imager was used for image capture.

First = First reactant added to 293 cells lysate

Second = Second reactant added to 293 cells lysate co-incubated with first reactant

I = Iodoacetamide N = N-Ethylmaleimide A = Acrylamide - = equivalent volume of deionized water was added X = Samples not included in the manuscript figures

Figure 5

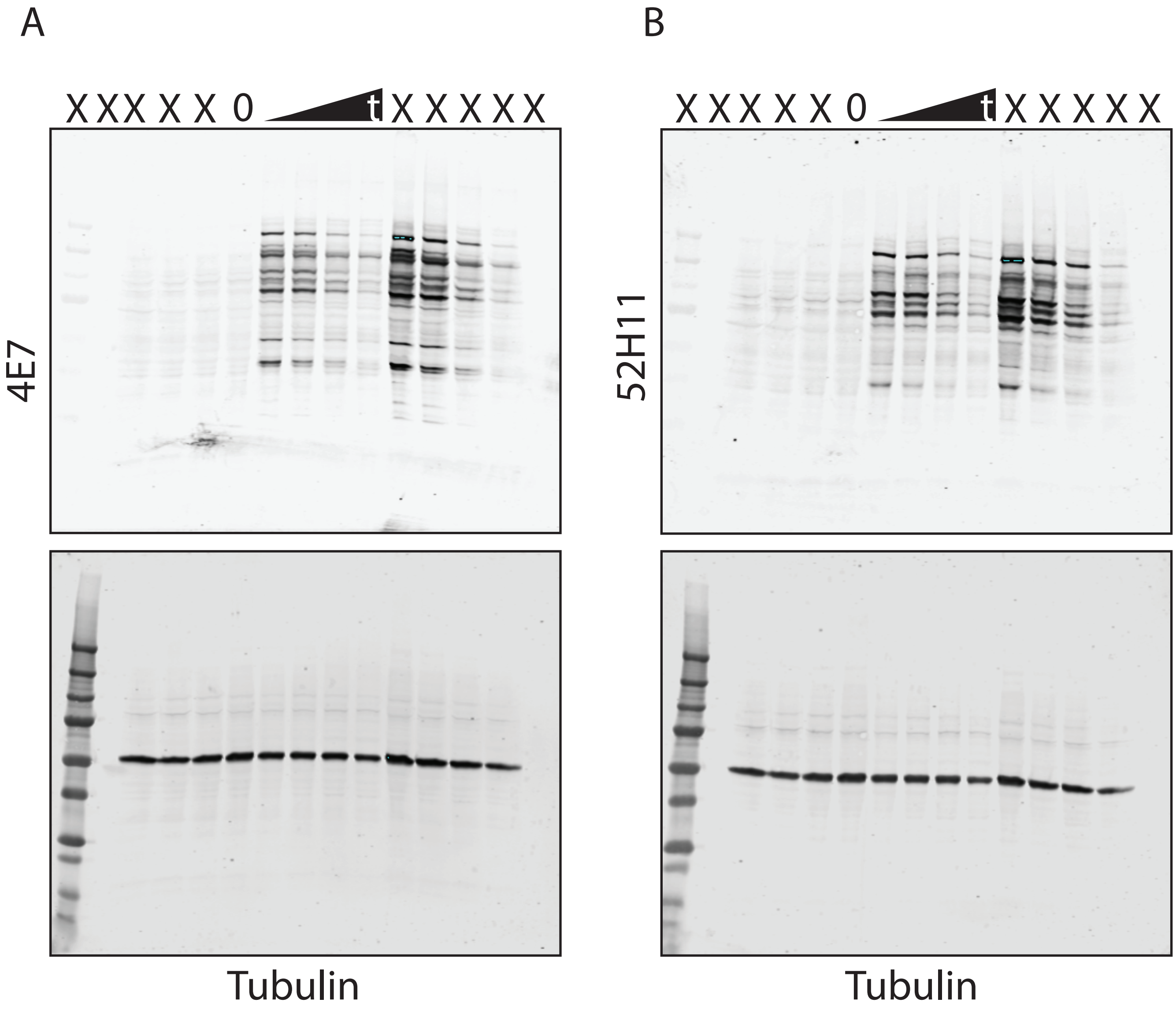

Samples were loaded as indicated in the figures above and Odyssey LICOR imager was used for image capture.

0 = no treatment with iodoacetamide

t = time after removal of iodoacetamide from the cells culture media

X = Samples not included in the manuscript figures.

Figure 6

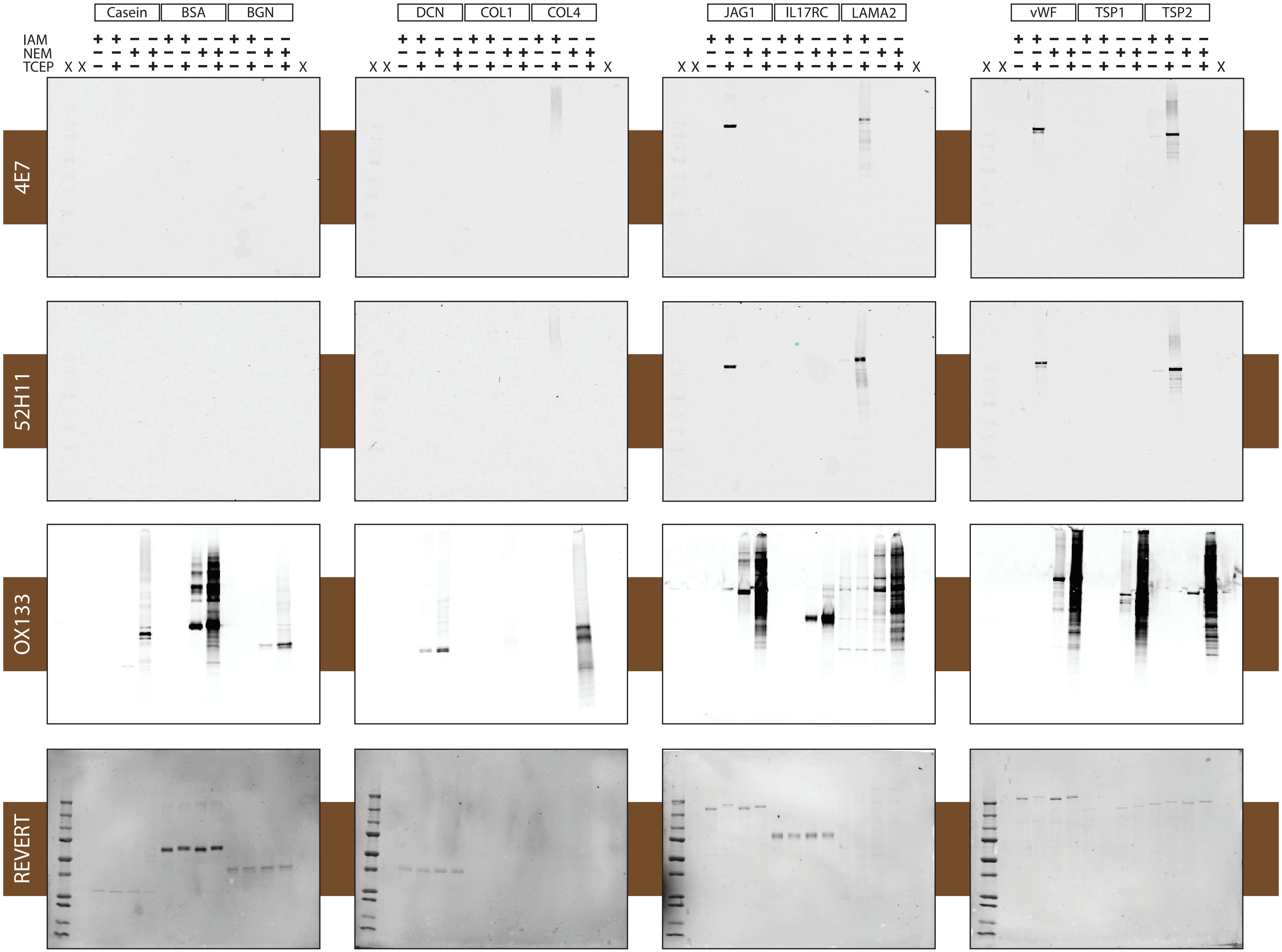

Samples were loaded as indicated in the figures above and Odyssey LICOR imager was used for image capture.
IAM = Iodoacetamide NEM = N-Ethylmaleimide TCEP = Tris (2-carboxyethyl) phosphine X = samples not included in manuscript figures

Figure 7

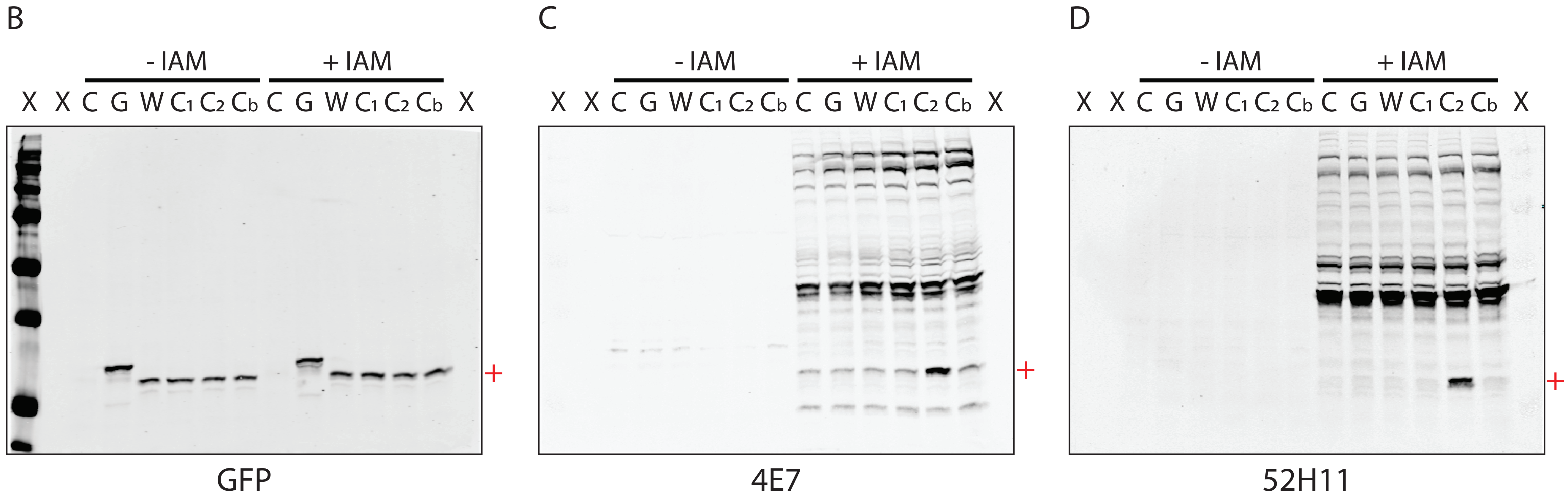

Samples were loaded as indicated in the figures above and Odyssey LICOR imager was used for image capture.
IAM = Iodoacetamide C = Control G = GFP W = Wild type human MYADML2 fragement
C_1, C_2, C_b = three varients with cysteine to serine mutation X = samples not included in the manuscript figures

Supplemental Figure 4

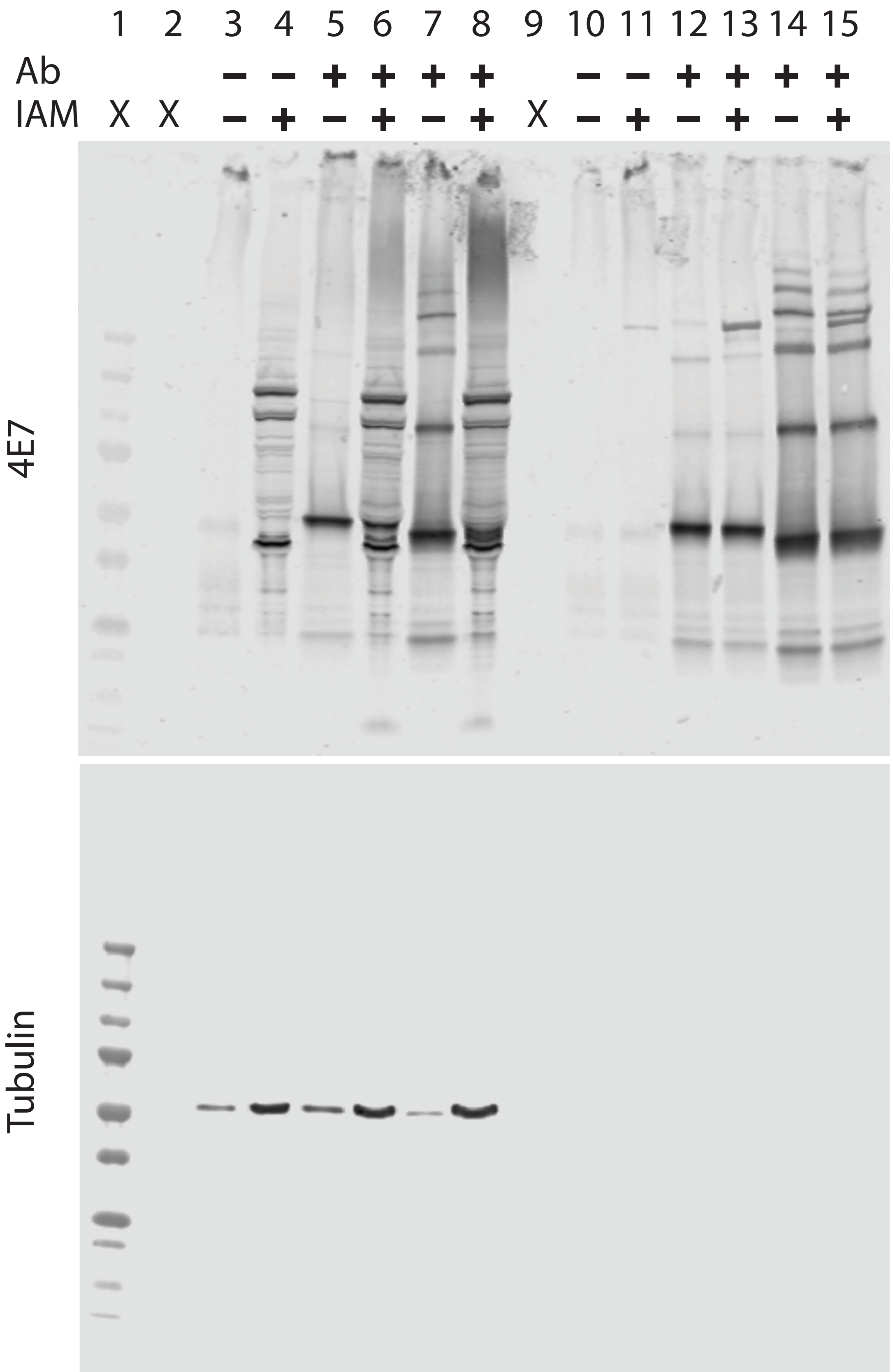

Samples were loaded as indicated in the figures and Odyssey LICOR imager was used for image capture.
IAM = Iodoacetamide
Ab = Anti-IAM antibodies, 4E7 (Lane 5, 6, 12, 13) or 52H11 (Lane 7, 8, 14, 15)
X = Samples not included in the manuscript figures
